# Role of defective calcium regulation in cardiorespiratory dysfunction in Huntington’s disease

**DOI:** 10.1172/jci.insight.140614

**Published:** 2020-10-02

**Authors:** Haikel Dridi, Xiaoping Liu, Qi Yuan, Steve Reiken, Mohamad Yehya, Leah Sittenfeld, Panagiota Apostolou, Julie Buron, Pierre Sicard, Stefan Matecki, Jérome Thireau, Clement Menuet, Alain Lacampagne, Andrew R. Marks

**Affiliations:** 1 Department of Physiology and Cellular Biophysics, Clyde and Helen Wu Center for Molecular Cardiology, Columbia University Vagelos College of Physicians and Surgeons, New York, New York, USA.; 2 PHYMEDEXP, University of Montpellier, CNRS, INSERM, CHRU Montpellier, Montpellier, France.; 3 Institut de Neurobiologie de la Méditerranée, INMED UMR1249, INSERM, Aix-Marseille Université, Marseille, France.; 4 LIA MusCaRyR, CNRS, Montpellier, France.

**Keywords:** Cell Biology, Therapeutics, Calcium channels, Calcium signaling, Drug therapy

## Abstract

Huntington’s disease (HD) is a progressive, autosomal dominant neurodegenerative disorder affecting striatal neurons beginning in young adults with loss of muscle coordination and cognitive decline. Less appreciated is the fact that patients with HD also exhibit cardiac and respiratory dysfunction, including pulmonary insufficiency and cardiac arrhythmias. The underlying mechanism for these symptoms is poorly understood. In the present study we provide insight into the cause of cardiorespiratory dysfunction in HD and identify a potentially novel therapeutic target. We now show that intracellular calcium (Ca^2+^) leak via posttranslationally modified ryanodine receptor/intracellular calcium release (RyR) channels plays an important role in HD pathology. RyR channels were oxidized, PKA phosphorylated, and leaky in brain, heart, and diaphragm both in patients with HD and in a murine model of HD (Q175). HD mice (Q175) with endoplasmic reticulum Ca^2+^ leak exhibited cognitive dysfunction, decreased parasympathetic tone associated with cardiac arrhythmias, and reduced diaphragmatic contractile function resulting in impaired respiratory function. Defects in cognitive, motor, and respiratory functions were ameliorated by treatment with a novel Rycal small-molecule drug (S107) that fixes leaky RyR. Thus, leaky RyRs likely play a role in neuronal, cardiac, and diaphragmatic pathophysiology in HD, and RyRs are a potential novel therapeutic target.

## Abstract 2

This study explores the role of ryanodine receptor calcium channels in the brain, the heart, and the diaphragm and central versus peripheral pathophysiological mechanisms in Huntington’s disease.

## Graphical abstract

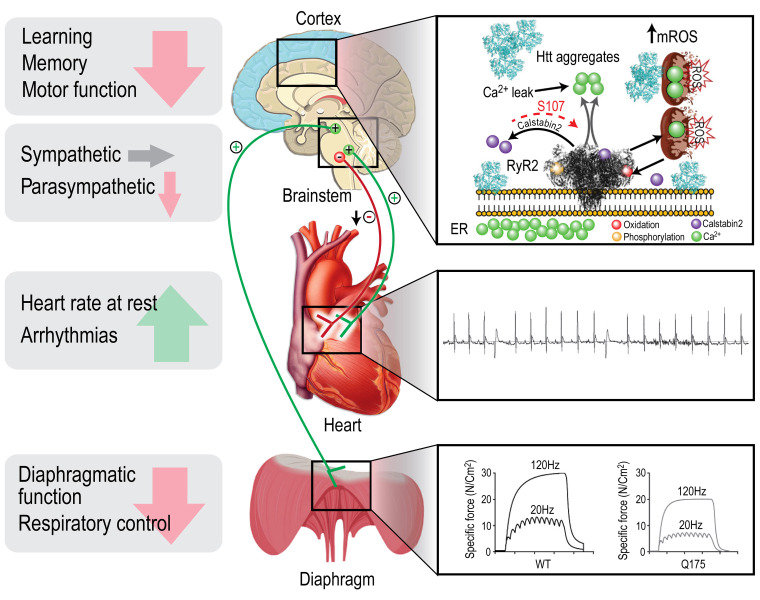

## Introduction

Huntington’s disease (HD) is a progressive, autosomal dominant neurodegenerative disorder affecting striatal neurons in young adults with distinct symptoms of cognitive decline and muscle incoordination (1, 2). This devastating disease is usually fatal approximately 10–15 years after onset of symptoms, and there is no disease-modifying treatment. HD is caused by a mutation in the HTT gene on chromosome 4 encoding the huntingtin protein. CAG repeat expansion results in a polyglutamine region (poly Q) at its N-terminus (3). Patients with HD exhibit impaired locomotor and respiratory muscle function and digestive tract dysfunction (4).

The leading cause of death in patients with HD is aspiration pneumonia (5, 6), due to dysphagia (7, 8). Indeed, diaphragmatic muscle weakness combined with difficulty clearing airway secretions and defective swallowing are prevalent in patients with HD (9, 10). However, the mechanisms underlying the respiratory dysfunction remain unknown (11–13). Most patients with HD do not report respiratory symptoms until later stages of the disease, when impaired swallowing and respiratory muscle weakness cause fatal aspiration pneumonias (7, 11, 14). At the late stages of HD, 44% of patients receive respiratory therapy compared with 2% at the early stages of the disease (12). In a recent study, patients with HD exhibited reduced respiratory pressure, forced vital capacity, peak expiratory flow, and maximal voluntary ventilation (13).

Heart failure occurs in approximately 30% of patients with HD (15), in contrast to less than approximately 2% of age-matched individuals in the general population (16–22). Despite the limited number of studies evaluating heart function in HD, epidemiological data identify cardiac disease as the second most common cause of death in HD. Recently Stephen et al. reported significant cardiac contractile dysfunction and ECG abnormalities in a large cohort of early symptomatic patients with HD; 25.3% of them exhibit ECGs abnormalities including bradycardia and prolonged Qtc interval (23).

Autonomic nervous system (ANS) dysfunction is a feature of HD and may play a role in the increased risk of cardiac arrhythmias in HD (4, 24, 25). Patients with HD may exhibit reduced heart rate variability (HRV) and altered sympathetic and parasympathetic activity (26, 27). Decreased cardiovagal regulation was found in patients with HD characterized by reduced HRV at rest and during deep respiration (24). It has been suggested that dysautonomia results in fatal cardiac arrhythmias (25, 28), due to impaired cardiac parasympathetic and sympathetic signaling (29). The cardiorespiratory consequences and possible mechanisms of ANS dysfunction in patients with HD are poorly understood. Ironically, patients with HD with cardiorespiratory manifestations are often excluded from clinical studies.

Neurodegeneration in HD may be associated with impaired synaptic transmission (29, 30), reduced brain-derived neurotrophic factor (31, 32), mitochondrial dysfunction (33, 34), and altered calcium (Ca^2+^) regulation (35–37). Increased intracellular Ca^2+^ concentration due to NMDA receptor activity or other Ca^2+^ sources including the inositol 1,4,5-trisphosphate receptor type 1 (IP3R1) and ryanodine receptor (RyR) may play a role in striatal neurodegeneration in HD (38–42).

RyRs are ubiquitous intracellular Ca^2+^ release channels expressed early in development (43) and required for the function of many organs including the heart, skeletal muscle, and synaptic transmission in the brain (44). Three RyR mammalian isoforms are known: RyR1 (45, 46), RyR2 (47), and RyR3 (48), which are classified as skeletal muscle, heart, and brain types, respectively (44), although all 3 forms are expressed in the brain. In skeletal muscle RyR activation is linked to voltage-gated calcium channels (49), and in other organs including the heart, RyRs are calcium-activated calcium release channels (44). RyRs are homotetrameric macromolecular protein complexes (50) that include 4 RyR protomers (565 kDa each), kinases (PKA, Ca2^+^/calmodulin kinase [CaMKII]), phosphatases (PP1, PP2A), calmodulin, and a phosphodiesterase (PDE4D3) (47). The RyR channel-stabilizing subunit calstabin (FKBP12) is critical for stabilizing the closed state of the channel and preventing a pathological leak of calcium (45, 51). Maladaptive cAMP-dependent kinase A–mediated (PKA-mediated) phosphorylation and redox-dependent modifications (cysteine nitrosylation and oxidation) (52–54) of RyRs have been linked to a loss of calstabin from the channel macromolecular complex (47, 55–57). RyR remodeling, in turn, results in impaired Ca^2+^ handling because of a pathological Ca^2+^ leak from the sarcoplasmic reticulum/ER (SR/ER) and is associated with multiple organ dysfunction, including cognitive impairment (54, 58), heart failure (59), and respiratory/locomotor muscle weakness (47, 58, 60–65).

Defective RyR function has been reported in HD, leading to elevated intracellular Ca^2+^ levels and reduced endoplasmic reticular Ca^2+^ stores in R6/2 striatal and cortical neurons (40). Moreover, RyR inhibitors have been shown to be neuroprotective in vitro and improve motor behavior in vivo in YAC128 mice (37, 40, 66).

Because defective Ca^2+^ regulation has been well documented in HD, but not understood mechanistically, we hypothesized that leaky neuronal RyR plays a role, especially in the cardiorespiratory pathology. We focused on the type 2 isoform of RyR (RyR2) because it is predominantly expressed in the brain (67). Indeed, we previously showed that oxidation, nitrosylation, and PKA phosphorylation of RyR2 results in leaky channels that contribute to the pathophysiology of posttraumatic stress disorder (54) and Alzheimer’s disease (AD) (58, 60). The role of RyR2 remodeling in brainstem nuclei remains unknown but might be involved in cardiac and respiratory dysfunction, such as arrhythmias and diaphragm muscle weakness in HD.

HD is characterized by cognitive dysfunction and involuntary motor movements (68, 69). We sought to determine whether leaky neuronal RyR2 channels play a role in cognitive dysfunction associated with HD. In addition, using a novel Rycal drug (S107), which crosses the blood-brain barrier and fixes the leak in RyR2 channels (54, 58, 70), we further determined whether inhibiting RyR2-mediated ER Ca^2+^ leak can improve cognitive function in a murine model of HD. S107 is a small molecule that stabilizes the RyR2-calstabin2 interaction and decreases RyR2 ER Ca^2+^ leak without affecting PKA phosphorylation, oxidation, and cysteine nitrosylation of the channel (54, 58, 60).

## Results

### Neuronal RyR2 channels are leaky in patients with HD and in a murine model of HD.

**Figure 1:**
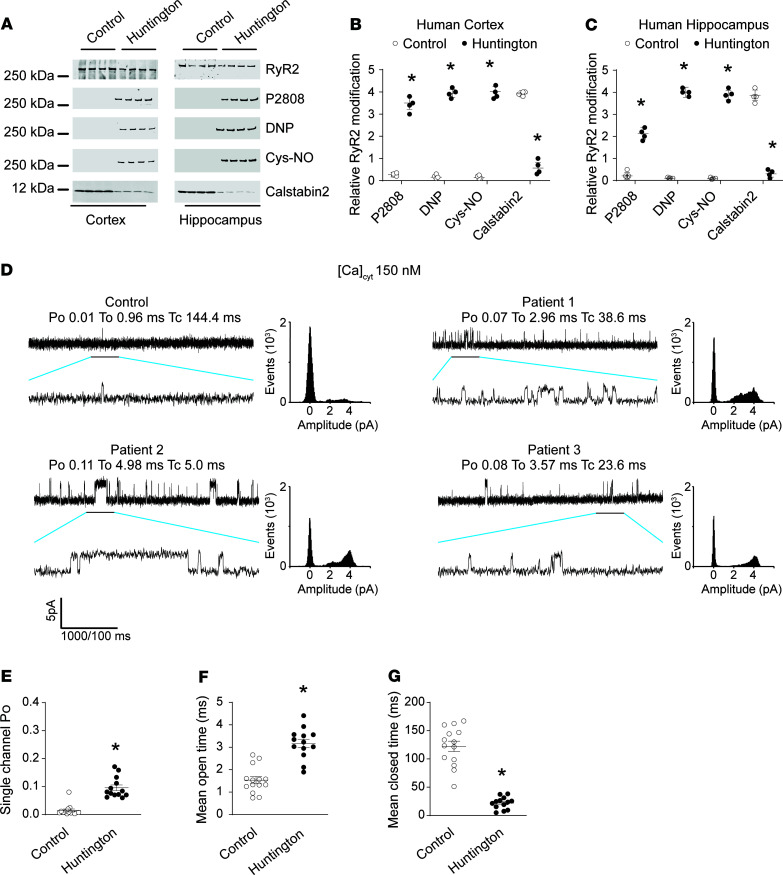
Cortex and hippocampal RyR2 channel remodeling result in the biochemical signature of leaky RyR2 in human HD brains. Representative SDS-PAGE analysis and quantification (bands normalized to total RyR2) of RyR2 immunoprecipitated from cortex (**A** and **B**) and hippocampus (**A** and **C**) from human samples (see Supplemental Table 1). Control (*n* = 4); patients with HD (*n* = 4) (band intensities were normalized to total RyR2). (**D**) Single-channel recordings of RyR2 incorporated in planar lipid bilayers with 150 nM Ca^2+^ in the cis chamber, corresponding to representative experiments performed with human cortex samples from controls and 3 patients with HD. RyR2 open probability (Po), mean open time (To) were increased, and the mean closed time (Tc) was decreased in HD RyR2 channels (**E**–**G**). P_O_ was 0.014 ± 0.005 in control (*n* = 14) and in patients with HD increased to 0.095 ± 0.01 (*n* = 13). Results are expressed as mean ± SD. Student’s *t* test; *P < 0.05, control vs. HD patient. Cys-NO, cysteine nitrosylation; DNP, 2,4-dinitrophenylhydrazine.

To evaluate the remodeling and functional abnormalities of RyR2 in the brains of HD patients, cortical and hippocampal biopsies from deidentified organ donors (Supplemental Table 1; supplemental material available online with this article; https://doi.org/10.1172/jci.insight.140614DS1) with neuropathological grade 3 and 4 HD, 5 female and 3 male (ages 54–66, CAG repeats 36–51), were obtained from the New York Brain Bank at Columbia University. We compared these specimens with cortical and hippocampal specimens from non-HD controls. ER fractions were purified to analyze the composition of the RyR2 macromolecular complex and posttranslational modifications known to be associated with channel leak (71) in the cortex and hippocampus (Figure 1, A–C). HD RyR2 exhibited PKA hyperphosphorylation (on RyR2-Ser2808), oxidation, and cysteine nitrosylation and were depleted of calstabin2 compared with controls. This is the “biochemical signature” of leaky RyR2 channels (47, 72). Single-channel recordings of cortical RyR2, reconstituted into planar lipid bilayers, revealed an increased open probability (P_O_) in the presence of low nonactivating [Ca^2+^]_cis_ conditions under which normal RyR2 channels are tightly closed (Figure 1, D–G). This elevated P_O_ is consistent with pathological ER Ca^2+^ leak (54, 58). Indeed, neuronal microsomes from HD cortex and hippocampus exhibited increased RyR-mediated ER Ca^2+^ leak compared with controls (Supplemental Figure 1, A–D).

We evaluated the time course (at 2, 6, and 10 months of age) of RyR2 remodeling in 3 regions of the brain (striatum, cortex, and hippocampus) using 2 HD murine models (Q111 and Q175) compared with controls. We observed RyR2 remodeling in a time-dependent manner with optimal onset of oxidation, phosphorylation, and calstabin2 depletion being at 10 months of age (Supplemental Figure 2).

**Figure 2:**
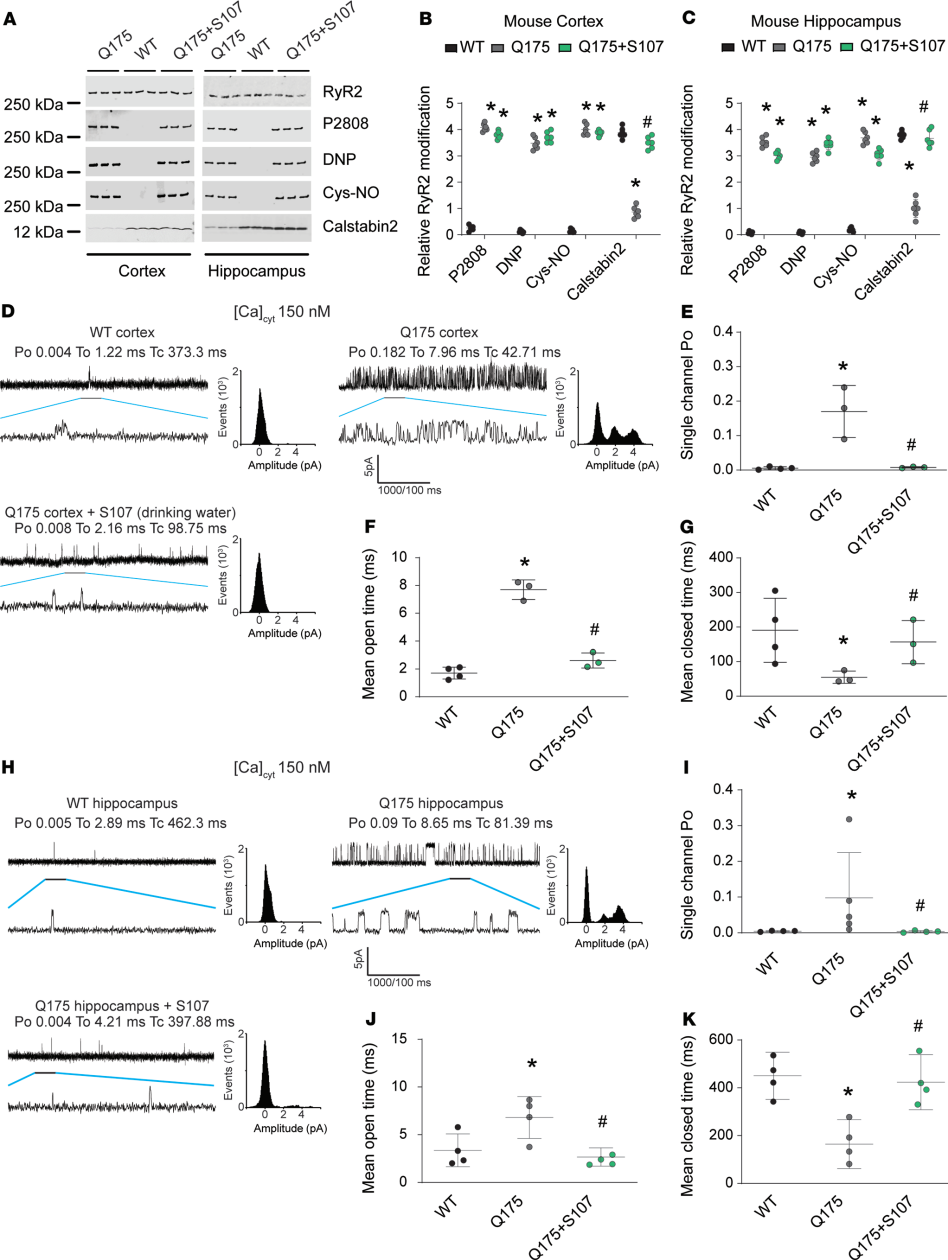
Cortical and hippocampal RyR2 channel remodeling results in the biochemical signature of leaky RyR2 in the brains of Q175 mice. Representative SDS-PAGE analysis and quantification of RyR2 immunoprecipitated from cortex (**A** and **B**) and hippocampus (**A** and **C**) from mouse samples. WT (*n* = 6), Q175 (*n* = 6), and Q175 (*n* = 6) treated with S107 (bands’ intensities were normalized to total RyR2). (**D**) Single-channel traces of RyR2 incorporated in planar lipid bilayers with 150 nM Ca^2+^ in the cis chamber, corresponding to representative experiments performed with mouse cortex samples from WT, Q175, and Q175 treated with S107. (**E**–**G**) RyR2 P_O_ was increased in HD. Mean P_O_ was 0.005 ± 0.002 in WT (*n* = 4) and in Q175 mice increased to 0.17 ± 0.04 (*n* = 3) and restored by S107 treatment to 0.008 ± 0.001 (*n* = 3). (**H**) Single-channel traces of RyR2 incorporated in planar lipid bilayers with 150 nM Ca^2+^ in the cis chamber, corresponding to representative experiments performed with mouse hippocampus samples from WT, Q175, and Q175 treated with S107. RyR2 P_O_ and T_O_ were increased and Tc was decreased in HD (**I**–**K**). P_O_ was 0.004 ± 0.0007 in WT (*n* = 4) and in Q175 mice increased to 0.09 ± 0.05 (*n* = 4) and restored by S107 treatment to 0.003 ± 0.001 (*n* = 4). Data (mean ± SD) analysis was performed by 1-way ANOVA. Bonferroni’s posttest revealed *P < 0.05 vs. WT, ^#^*P* < 0.05 vs. Q175.

Then we chose the Q175 mouse model at 10 months old to evaluate RyR2 remodeling with and without S107 treatment compared with WT controls (littermates). As in HD patients’ samples, RyR2 exhibited the biochemical signature of leaky channels in the cortex and in the hippocampus of Q175 mice compared with WT. RyR2 was PKA phosphorylated on Ser2808, oxidized, cysteine nitrosylated, and depleted of the stabilizing subunit calstabin2 (Figure 2, A–C). This RyR2 remodeling was associated with evidence of leaky RyR2 channels based on increased P_O_ recorded at low nonactivating [Ca^2+^]_cis_ in both the cortex and hippocampus of Q175 mice (Figure 2, D–K). Indeed, isolated brain mitochondria from Q175 mice exhibited increased reactive oxygen species (ROS) production, which may explain in part the oxidation of RyR2 channels (Supplemental Figure 3A). S107 administered in the drinking water reduced calstabin2 dissociation from the RyR2 macromolecular complex and decreased ER Ca^2+^ leak (Figure 2, A–K).

### Leaky RyR2 causes cognitive impairment in a murine model of HD.

**Figure 3:**
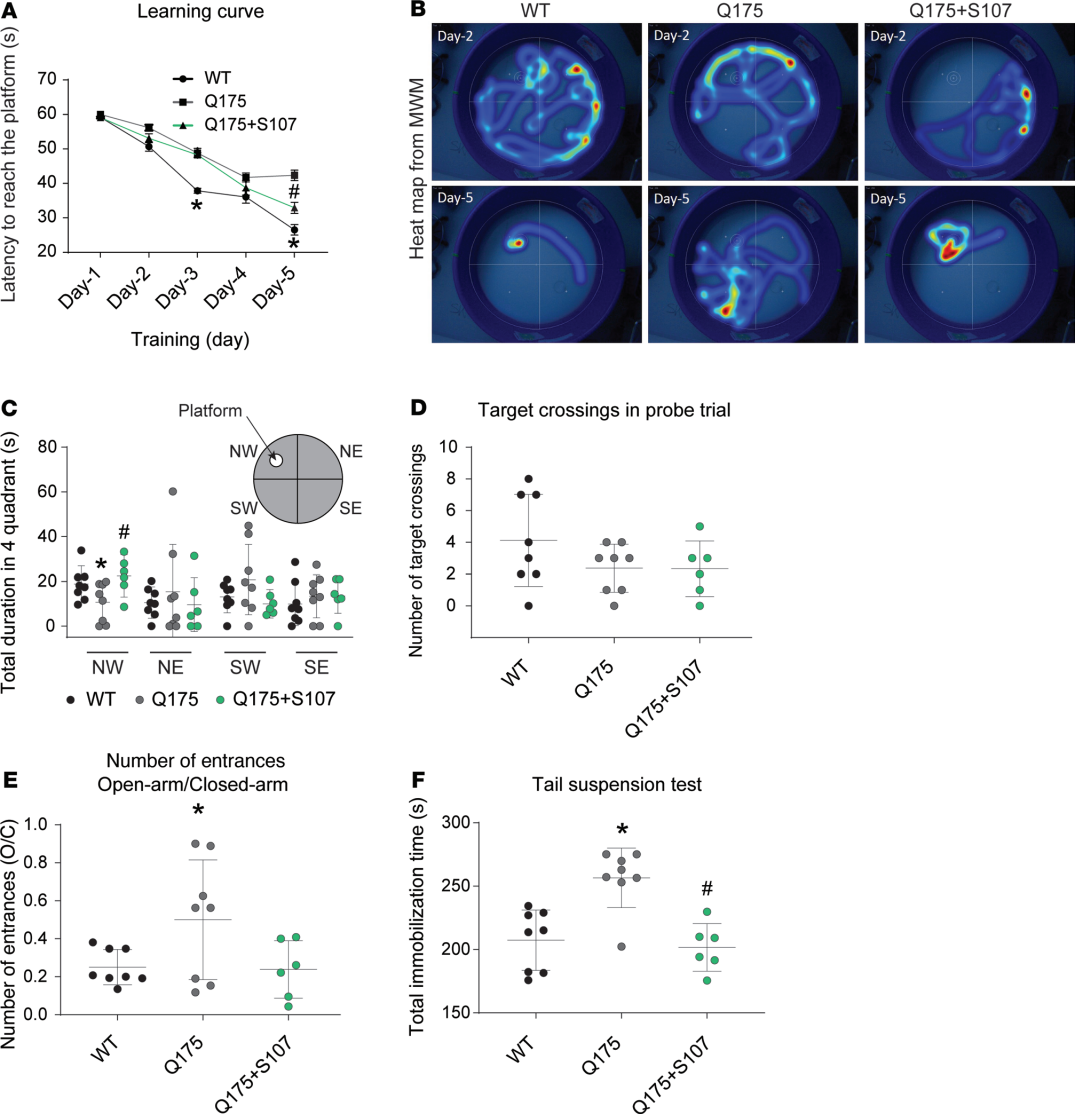
Long-term learning and memory deficits in Q175 mice. S107 treatment targeting RyR2 channels improves cognitive function in HD. (**A**) Learning curves showing the escape latency during a 5-day training period in the Morris water maze (MWM). (**B**) The heatmaps from all the training trials were recorded (Noldus Information Technology Inc.) and downloaded. Representative heatmaps from WT (left column), Q175 (middle column), and S107-treated Q175 (right column) on day 2 (upper panels) and day 5 (lower panels) are shown. (**C**) The time spent in all quadrants. (**D**) The number of target crossings on day 6 probe trial of the MWM. (**E**) The ratio of the number of entries to open arms versus closed arms of the elevated plus maze (EPM). (**F**) Total immobilization time of mice during 300 seconds of tail suspension test. The same groups of mice were used for MWM, EPM, and tail suspension. WT (*n* = 8), Q175 (*n* = 8), and Q175+S107 (*n* = 6). Data (mean ± SD) analysis was performed by 1-way ANOVA. Bonferroni’s posttest revealed *P < 0.05 vs. WT, ^#^*P* < 0.05 vs. Q175.

We assessed the effects of leaky RyR2 channels on spatial learning and memory using the Morris water maze (MWM) as previously described (54, 58). Q175 mice exhibited increased escape latency in the MWM at days 3 and 5 (Figure 3, A and B), spent less time in the target quadrant, and had a slightly reduced number of target crossings compared with WT mice (Figure 3, C and D). These findings are consistent with deficits in learning and/or memory. S107 treatment of Q175 mice significantly improved the escape latency and the time spent in the target quadrant compared with untreated Q175 mice, suggesting that RyR2 channel leak likely contributes to the cognitive deficits observed in the Q175 mice. Leaky RyR2 channels were also associated with increased anxiety determined using the elevated plus maze (EPM) test. Q175 mice spent more time and made more entries into the open arms of the EPM (Figure 3E). In the tail suspension test, Q175 mice exhibited increased immobilization duration consistent with increased stress, which was normalized by treatment with S107 (Figure 3F). The Q175 mice exhibited significantly impaired coordination in movement, suggesting deficits of motor neuron function. S107 treatment significantly improved the dysregulated movement coordination. Kyphosis testing and gait analysis did not reach significance, suggesting that the impaired motor neuron function was moderate (Supplemental Table 2). To confirm our results, we evaluated the RyR2 remodeling and the subsequent cognitive dysfunction in a second murine model of HD (R/6 mice) and observed similar results (Supplemental Figure 4 and Supplemental Table 3).

### RyR2 phosphorylation in autonomic brainstem areas affecting the heart.

Although described as a disease of the central nervous system, studies have revealed abnormalities in non-neuronal organs in patients with HD (4). Whether these defects are a direct consequence of mutant huntingtin protein, or secondary to neurological dysfunction, is poorly understood. Cardiac function is regulated centrally via both parasympathetic and sympathetic activities. Cardiac vagal preganglionic neurons are located in the dorsal motor nucleus of the vagus (DMNV) and in the nucleus ambiguus (NA) in the medulla oblongata. A major source of cardiac sympathetic drive comes from adrenergic presympathetic C1 neurons located in the rostral ventrolateral medulla (73). C1 neurons are hyperactivated in multiple cardiovascular diseases, including heart failure, which might contribute to their etiology.

**Figure 4:**
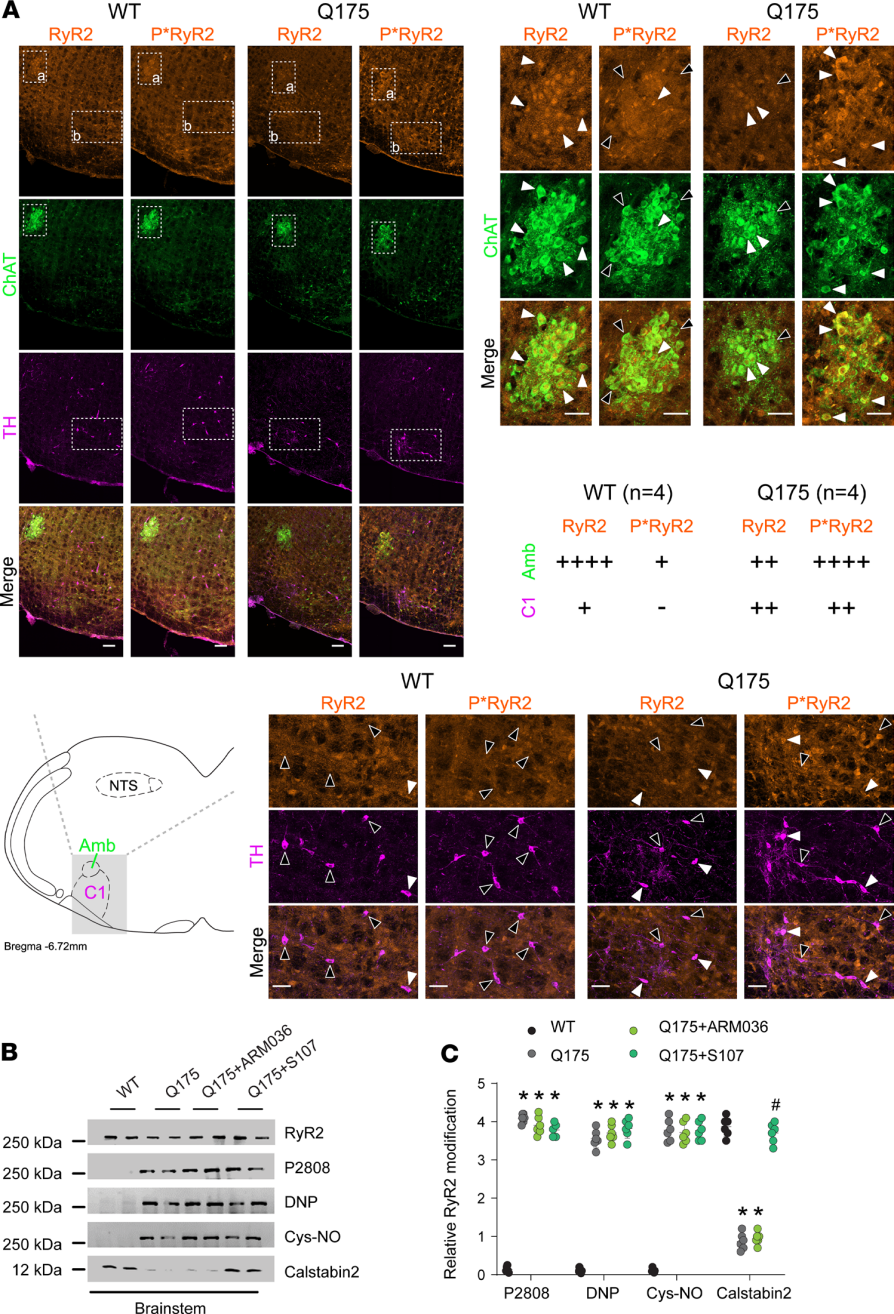
Brainstem RyR2 remodeling induces NA disruption. (**A**) Immunostaining and semiquantitative analysis of total and phosphorylated RyR2 (P*RyR2) on Ser2808 in NA neurons (choline acetyltransferase positive, ChAT positive), which include cardiac parasympathetic neurons, and in presympathetic rostral ventrolateral medulla C1 neurons (tyrosine hydroxylase positive, TH positive) in the brainstems of WT and Q175 mice. White arrows show ChAT/TH neurons with RyR2/P*RyR2 colocalization; black arrows show ChAT/TH neurons without RyR2/P*RyR2 colocalization. Semiquantitative analysis of ChAT/TH and RyR2/P*RyR2 colocalization were made as follows: - = 0% costaining, + = 1% to 25% costaining, ++ = 26% to 50% costaining, +++ = 51% to 75% costaining, and ++++ = 76% to 100% costaining. Analysis was made in 4 WT and 4 Q175 mice, bilaterally in 2–3 sections per animal. (**B** and **C**) Representative SDS-PAGE analysis and quantification of RyR2 immunoprecipitated from the brainstems collected from WT (*n* = 6), Q175 (*n* = 6), Q175+ARM036 (*n* = 6), and Q175+S107 (*n* = 6) mice (band intensities were normalized to total RyR2). Data (mean ± SD) analysis was performed by 1-way ANOVA. Bonferroni’s posttest revealed *P < 0.05 vs. WT, ^#^*P* < 0.05 vs. Q175. Scale bars: 100 μm (top left), 50 μm (top right and middle right). Amb, nucleus ambiguus; NTS, nucleus tractus solitarius.

**Figure 5:**
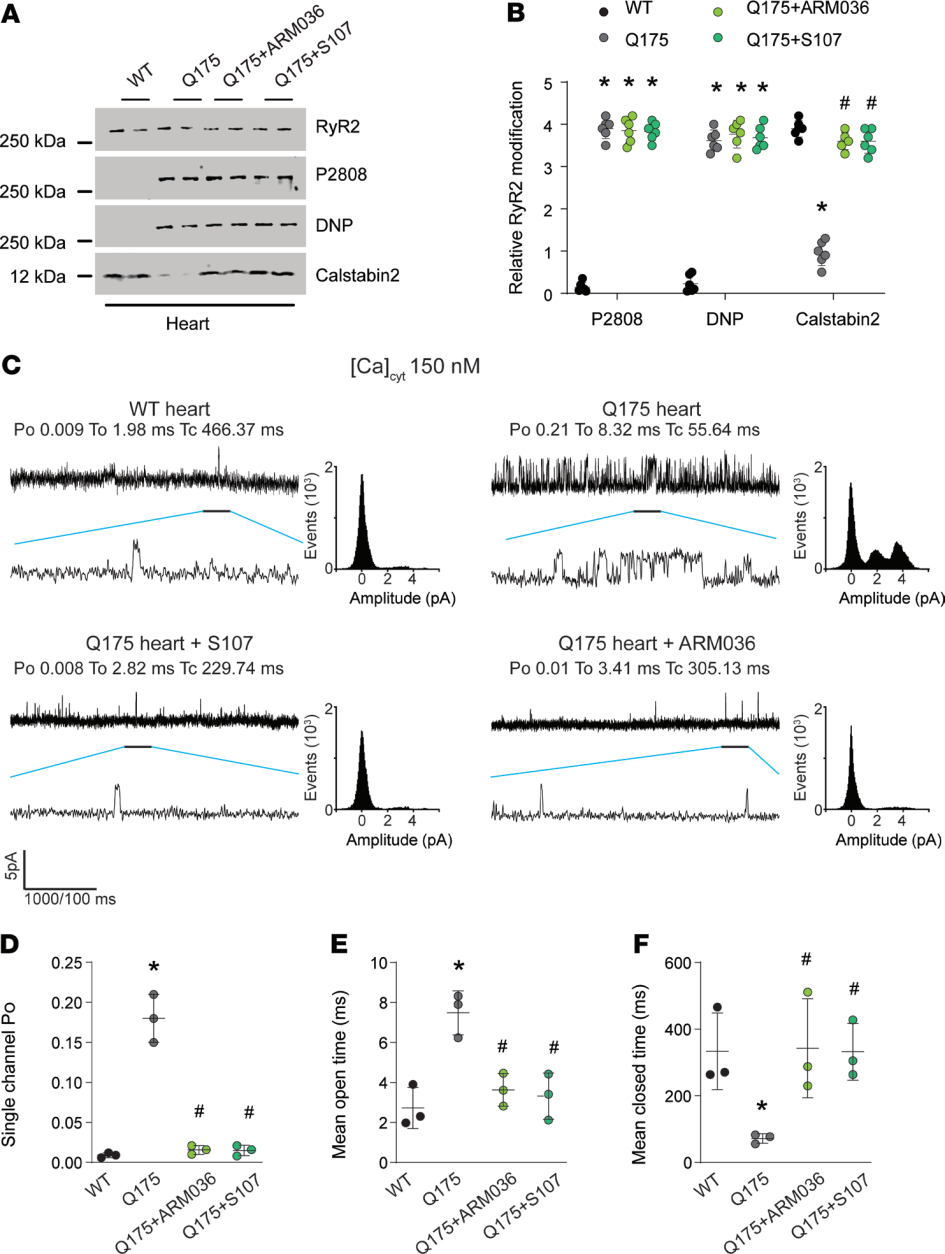
Cardiac RyR2 remodeling in Q175 mice. (**A** and **B**) Representative SDS-PAGE analysis and quantification of RyR2 immunoprecipitated from the heart samples from WT (*n* = 6), Q175 (*n* = 6), Q175+ARM036 (*n* = 6), and Q175+S107 (*n* = 6) (bands’ intensities were normalized to total RyR2). (**C**) Single-channel traces of RyR2 incorporated in planar lipid bilayers with 150 nM Ca^2+^ in the cis chamber, corresponding to representative experiments performed with heart samples from WT, Q175, Q175+ARM036, and Q175+S107 mice. (**D**–**F**) Single-channel analysis: P_O_ was 0.009 ± 0.001 in WT (*n* = 3) and in Q175 mice increased to 0.18 ± 0.01 (*n* = 3) and restored by S107 treatment to 0.015 ± 0.003 (*n* = 3) and ARM036 to 0.015 ± 0.003 (*n* = 3). Data (mean ± SD) analysis was performed by 1-way ANOVA. Bonferroni’s posttest revealed *P < 0.05 vs. WT, ^#^*P* < 0.05 vs. Q175.

We evaluated the levels of RyR2 phosphorylation in NA and C1 neurons by immunohistochemistry in HD mice. Choline acetyltransferase and tyrosine hydroxylase staining were used as markers of NA and C1 neurons, respectively. Compared with WT, there was increased RyR2 phosphorylation at Ser2808 in the NA (Figure 4A). There was also increased RyR2 PKA phosphorylation in C1 neurons, to a lesser extent than in the NA (Figure 4A). This finding was confirmed by immunoprecipitation. Brainstem RyR2 exhibited increased Ser2808 phosphorylation, oxidation, cysteine nitrosylation, and depletion of the stabilizing subunit calstabin2 (Figure 4, B and C). We treated the HD mice with S107 (blood-brain barrier [BBB] permeant) and ARM036 (BBB nonpermeant) Rycals to understand the link between brainstem RyR2 abnormalities and autonomic dysfunction. S107 treatment, but not ARM036, prevented calstabin2 dissociation from the RyR2 macromolecular complex in the brainstem, demonstrating a central origin of these defects. In addition, cardiac RyR2 exhibited increased Ser2808 phosphorylation, oxidation, cysteine nitrosylation, and depletion of the stabilizing subunit calstabin2 (Figure 5, A and B). This cardiac RyR2 remodeling was associated with a significant increase in RyR2 P_O_ in heart samples from Q175 mice consistent with leaky channels (Figure 5, C–F). Both S107 and ARM036 prevented calstabin2 dissociation from cardiac RyR2.

### Remodeled RyR2 in brainstems contributes to altered HRV and cardiac arrhythmias in a murine model of HD.

**Figure 6:**
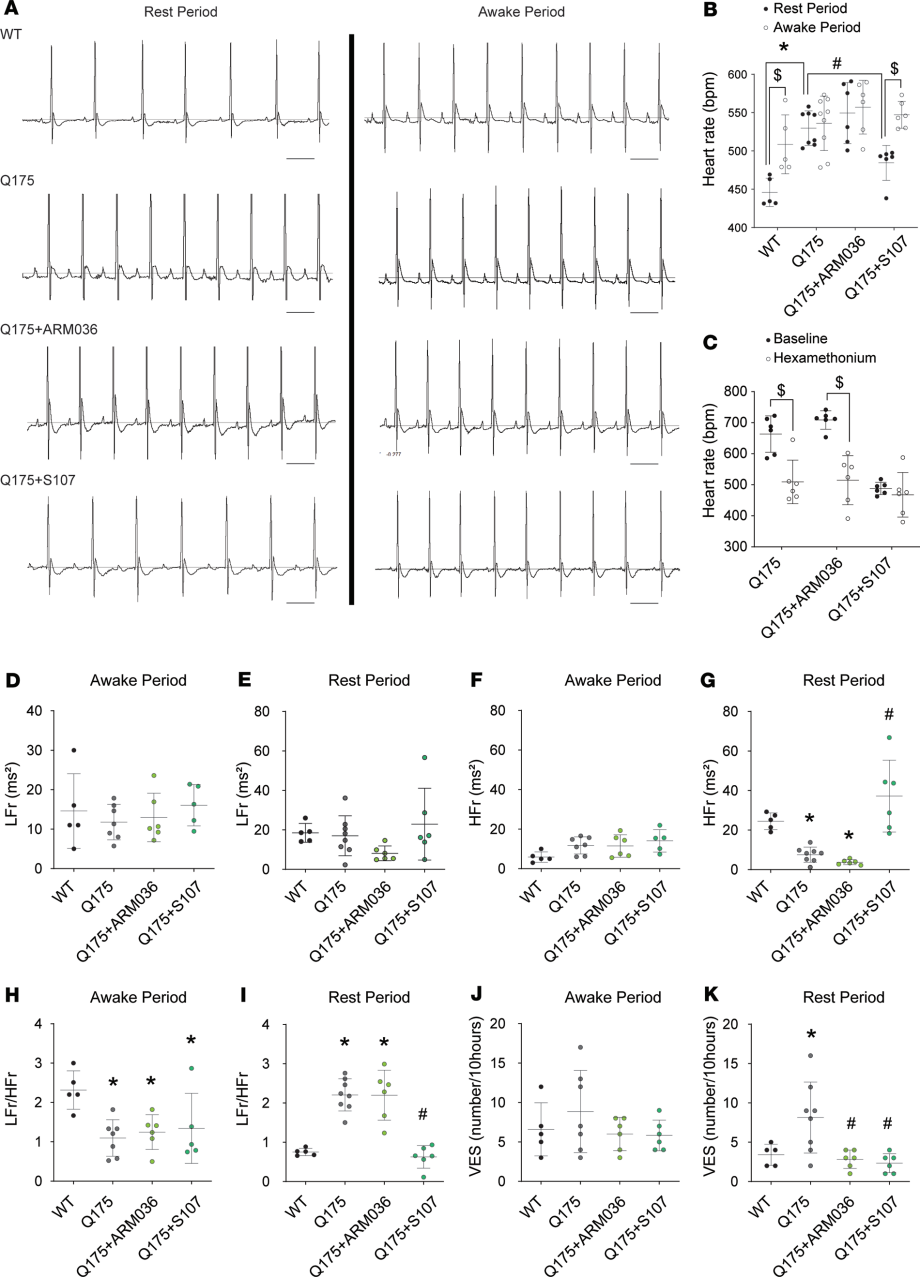
Sympathovagal imbalance and arrhythmias in HD during rest period. (**A**) Representative ECG trace in freely moving conscious animals allowing HR (bpm) measurement in WT and Q175 mice treated or not with S107 or ARM036 during light (rest period) and dark (awake period) cycles. (**B**) HR (bpm) average during awake and rest periods in WT (*n* = 5), Q175 (*n* = 8), and Q175 mice treated or not with S107 (*n* = 6) or ARM036 (*n* = 6). (**C**) HR average record. Hexamethonium injection (20 mg/kg) had moderate effects on HR related to suppression of cardiac autonomic control of HR, that is, lowering HR during the daylight period in Q175 (*n* = 6) and Q175 mice treated or not with S107 (*n* = 6) or ARM036 (*n* = 6). Data (mean ± SD) analysis was performed by 1-way ANOVA. Bonferroni’s posttest revealed *P < 0.05 vs. WT, ^#^*P* < 0.05 vs. Q175 at rest period. Student’s *t* test, ^$^*P* < 0.05 rest vs. awake period and baseline vs. hexamethonium. (**D** and **E**) Low-frequency (LFr) spectral power density measured by HRV analysis using fast Fourier transformation (LFr: 0.15–1.5 Hz) in WT, Q175, Q175+ARM036, and Q175+S107 during rest and awake periods (*n* = 5–8 mouse/group). (**F** and **G**) High-frequency (HFr) spectral power measured by HRV analysis using fast Fourier transformation (HFr: 1.5–5 Hz) during rest and awake periods in WT, Q175, Q175+ARM036, and Q175+S107 (*n* = 5–8 mouse/group). (**H** and **I**) LFr/HFr ratio (*n* = 5–8 mouse/group). (**J** and **K**) Number of isolated and triplet (3 consecutive) ventricular extrasystoles (VESs) during 10 hours in WT, Q175, Q175+ARM036, and Q175+S107 during rest and awake periods (*n* = 5–8 mouse/group). Comparison of HRV (standard deviation of NN intervals) and VESs at rest versus awake is shown in Supplemental Figure 5, D–F. Representative example of VESs in Q175 mice is shown in Supplemental Figure 5G. Data (mean ± SD) analysis was performed by 1-way ANOVA. Bonferroni’s posttest revealed *P < 0.05 vs. WT, ^#^*P* < 0.05 vs. Q175. Scale bar: 110 ms.

To evaluate the ANS outflows, we monitored cardiac electric activity (ECG) using a telemetric system over 24 hours in freely moving, conscious WT, Q175, and Q175 animals treated with S107 or ARM036. Heart rate (HR) is controlled by the activity of the pacemaker and modulated by the sympathetic and parasympathetic limbs of the ANS. We hypothesized that the RyR2 remodeling in NA and C1 neurons could induce a sympathovagal imbalance contributing to cardiac arrhythmias. During the awake period, we observed a slight but not significant increase in HR in Q175 mice (Figure 6, A and B). During the rest period, HR was significantly increased in Q175 mice compared with WT, suggesting a reduction in the parasympathetic tone during rest in line with the NA and RyR2 remodeling observed in the brainstem slices. Moreover, fixing the calcium leak with S107 but not ARM036 significantly reduced HR at rest (Figure 6, A and B).

Hexamethonium, which blocks both sympathetic and parasympathetic limbs of the ANS (74), caused a significant reduction in HR in both Q175 and Q175+ARM036 mice. These data suggest that the increased HR observed in Q175 mice was driven by decreased parasympathetic activity and/or increased sympathetic activity (Figure 6C).

We then analyzed the activity of the ANS manifested by beat-to-beat HRV (Figure 6, D–I). The low-frequency bands reflect mostly sympathetic modulation of heart rhythm, and oscillations in high-frequency bands reflect exclusively parasympathetic vagal activity. Thus, the low frequency/high frequency ratio reflects sympathovagal balance (75). The low frequency was similar between all the groups during active and rest periods, suggesting that sympathetic regulation of cardiac activity was normal in the Q175 HD mouse model. In contrast, there was a significant decrease in the high frequency during the rest period in Q175 mice compared with WT consistent with impaired parasympathetic activity. Q175 mice exhibited a lower low frequency/high frequency ratio compared with WT during the active period and a higher low frequency/high frequency ratio during the rest period (Figure 6, H and I). These data suggest that RyR2-mediated ER Ca^2+^ leak in the brainstem alters sympathovagal balance, through a reduction in parasympathetic activity that may promote cardiac arrhythmias. Indeed, ventricular extrasystoles (VESs) were increased in Q175 mice during the rest period but did not change during the awake period. The increased number of extrasystoles was abolished by both S107 and ARM036, suggesting that these VESs were triggered in the heart by diastolic SR Ca^2+^ leak as previously described (76). The remodeling of RyR2 in the heart described above accounts for diastolic SR Ca^2+^ leak and subsequent VES (Figure 6, J and K). Next, we evaluated cardiac function using ECG to measure the left ventricular ejection fraction (LVEF) and speckle tracking imaging that allows earlier detection of left ventricular systolic dysfunction, a strong predictor of heart failure and mortality (77). The LVEF in Q175 mice was normal; however, the left ventricular global longitudinal strain was reduced, indicating a predisposition to heart failure (Supplemental Figure 5, A–C).

### Remodeled leaky RyR2 in brainstems contributes to respiratory dysfunction.

Respiratory dysfunction including chest muscle rigidity, respiratory muscle weakness, difficulty in clearing airway secretions, and swallowing abnormalities have been reported in patients with neurodegenerative disorders, including HD (9, 10).

We evaluated ex vivo and in vivo the respiratory function in Q175 HD mice. In order to distinguish between centrally mediated versus peripherally mediated effects on respiratory function, we used 2 Rycal drugs that fix leaky RyR channels, S107 and ARM036.

**Figure 7:**
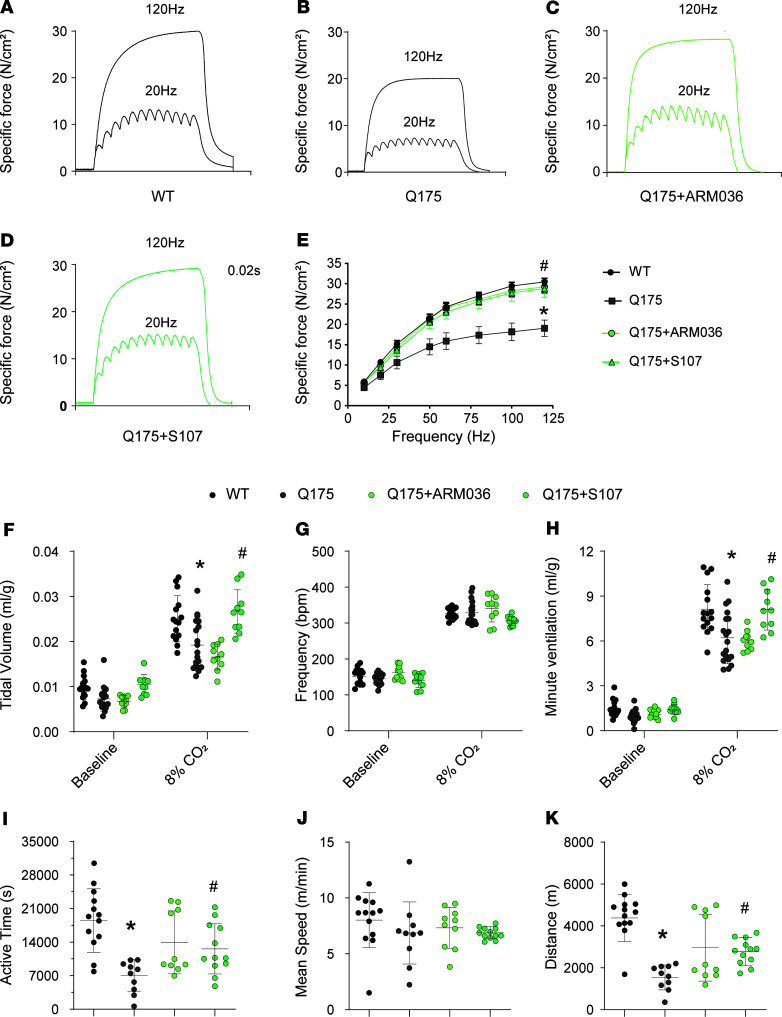
RyR-mediated SR Ca^2+^ leak contributes to respiratory dysfunction and reduces voluntary activity in a murine model of HD. (**A**–**D**) Representative records of diaphragmatic specific force production measured ex vivo at 20 and 120 Hz in muscle bundles under isometric conditions in WT, Q175, Q175+ARM036, and Q175+S107 mice. (**E**) Average force-frequency relationship recorded in WT (*n* = 13), Q175 (*n* = 10), Q175+ARM036 (*n* = 9), and Q175+S107 (*n* = 8) mice. Data (mean ± SEM) analysis was performed by 2-way ANOVA. Bonferroni’s posttest revealed *P < 0.05 vs. WT, ^#^*P* < 0.05 Q175 vs. Q175+S107/ARM036. (**F**) Tidal volume (mL/g) at rest and during CO_2_ stimulation. (**G**) Respiratory frequency (bpm) at rest and during CO_2_ stimulation (**H**) Minute volume (mL/g) at rest and during CO_2_ stimulation in WT (*n* = 15), Q175 (*n* = 19), Q175+ARM036 (*n* = 10), and Q175+S107 (*n* = 10). The CO_2_ values were recorded 10 minutes after the initiation of CO_2_ stimulation. Data (mean ± SD) analysis was performed by 1-way ANOVA. Bonferroni’s posttest revealed *P < 0.05 vs. WT, ^#^*P* < 0.05 vs. Q175. (**I**–**K**) Active time (seconds), mean speed (m/min), and running distance (m) for voluntary activity on running wheels, respectively, in WT (*n* = 13), Q175 (*n* = 10), Q175+ARM036 (*n* = 10), and Q175+S107 mice (*n* = 12). Data (mean ± SD) analysis was performed by 1-way ANOVA. Bonferroni’s posttest revealed *P < 0.05 vs. WT, ^#^*P* < 0.05 vs. Q175.

To evaluate the intrinsic properties of the respiratory muscle, we compared the ex vivo diaphragmatic function in Q175 versus WT mice. Force production was significantly reduced (Figure 7, A–E) at different stimulation frequencies (*P* < 0.05) in Q175 mice compared with their control littermates. Both S107 and ARM036 treatments restored diaphragmatic force generation in Q175 mice (Figure 7, C–E). Interestingly, extensor digitorum longus (EDL) and soleus contractile function were not affected in HD mice (Supplemental Figure 5, H–Q), suggesting that the muscle dysfunction was more severe in the diaphragm. One possibility is that the severity of the muscle dysfunction in the diaphragm was due to defective central regulation of respiratory function.

We used whole-body plethysmography to compare the respiratory function of Q175 versus WT mice with room air or hypercapnia (8% CO_2_) (Figure 7, F–H). With room air WT and Q175 mice had equivalent tidal volumes (TVs), respiratory frequency (F), minute ventilation (MV), and inspiration and expiration times (Figure 7, F–H; and Supplemental Figure 5, P–R). With hypercapnia (8% CO_2_), Q175 mice exhibited significantly reduced MV compared with the WT mice and decreased TV (*P* < 0.05) (Figure 7, F and H).

S107 and ARM036 administration had no effect on respiratory function in mice breathing room air (Figure 7, F–H). In contrast, S107 treatment, but not ARM036, rescued respiratory function in Q175 mice under hypercapnia. These data suggest that leaky neuronal RyR2 alters central respiratory control in Q175 HD mice. In addition, voluntary activity as determined using running wheel was significantly reduced in terms of time spent on the wheel in Q175 mice compared with control without any difference in the mean speed (Figure 7, I–K). Both S107 and ARM036 treatments similarly rescued the decreased voluntary activity observed in our HD mice.

### Diaphragmatic dysfunction in HD is associated with impaired excitation contraction coupling.

**Figure 8:**
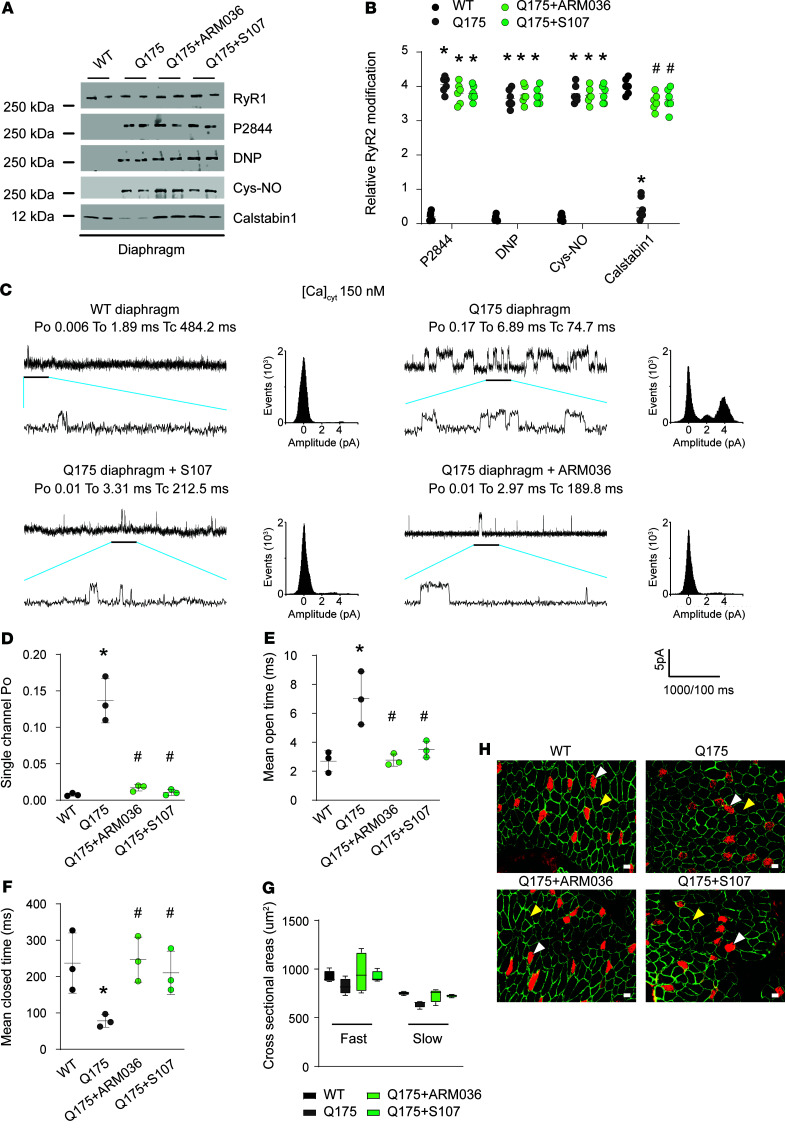
Diaphragmatic RyR1 remodeling in HD. (**A** and **B**) Representative SDS-PAGE analysis and quantification of RyR1 immunoprecipitated from diaphragm samples (band intensities were normalized to total RyR1) of WT (*n* = 6), Q175 (*n* = 6), Q175+ARM036 (*n* = 6), and Q175+S107 mice (*n* = 6). (**C**) Single-channel traces of RyR1 incorporated in planar lipid bilayers with 150 nM Ca^2+^ in the cis chamber, corresponding to representative experiments performed with diaphragm samples from Q175 mice. (**D**–**F**) Increased RyR1 P_O_ and T_O_ and decreased Tc in Q175 mouse diaphragms. P_O_ was 0.007 ± 0.001 in WT (*n* = 3) and in Q175 increased to 0.13 ± 0.017 (*n* = 3) and restored to 0.017 ± 0.02 in Q175+S107 (*n* = 3) and to 0.010 ± 0.002 in Q175+ARM036 (*n* = 3). Data (mean ± SD) analysis was performed by 1-way ANOVA. Bonferroni’s posttest revealed *P < 0.05 vs. WT, ^#^*P* < 0.05 vs. Q175. (**G**) Representative immunostaining of fast and slow diaphragm muscle fibers of WT (*n* = 6), Q175 (*n* = 6), Q175+ARM036 (*n* = 6), and Q175+S107 mice (*n* = 6). Quantified data are represented as a box-and-whisker plot, with bonds from 25th to 75th percentile, median line, and whiskers ranging from minimum to maximum values. Antibodies against fast-type (yellow arrows) and slow-type (white arrows) myosin ATPase were used to perform immunostaining on cryosections of mouse diaphragms. Muscle membrane was counterstained with dystrophin antibodies (green color). (**H**) Quantification of cross-sectional area (in μm^2^) was calculated using ImageJ software (NIH) in each condition. Scale bar: 50 μm.

Diaphragmatic dysfunction can occur in response to increased beta-adrenergic signaling, which causes PKA-mediated RyR1 hyperphosphorylation and leaky channels (78). RyR1 Ser2844 phosphorylation, oxidation, and cysteine nitrosylation were increased and calstabin1 was depleted from RyR1 in Q175 diaphragms (Figure 8, A and B). RyR1 remodeling was associated with a significant increase in RyR1 P_O_ under nonactivating conditions ([Ca^2+^]_cyt_ = 150 nM), consistent with leaky channels, which were fixed by treatment with either S107 or ARM036 (Figure 8, C–F). Moreover, diaphragmatic dysfunction was not due to reduced cross-sectional area (Figure 8, G and H).

## Discussion

Calcium is a second messenger that regulates activity of numerous cellular processes, including activation of protein kinases and phosphatases, proteases, ion transporters and channels, neurotransmitter vesicle release, and gene transcription. Ca^2+^ is stored in the endo/sarcoplasmic reticulum compartment and can be released via IP3Rs and/or RyRs (47, 79). In pathological conditions, remodeled RyR channels have been shown to mediate ER/SR Ca^2+^ leak into the cytosol at rest, leading to local increases of [Ca^2+^]_cyt_ and activation of pathological signals (60). In neurons, such increases in [Ca^2+^]_cyt_ can alter synaptic plasticity, survival/growth, and other essential signals (80). Interestingly, it has been shown in R6/2 HD mice that increased [Ca^2+^]_cyt_ is linked to locomotor dysfunction (81).

In the present study we show that RyR2 channels in the brain are oxidized, PKA phosphorylated, nitrosylated, and depleted of the stabilizing subunit calstabin in both human patients with HD and in 3 models of HD (R6/2, Q111, and Q175). This biochemical signature of leaky RyR2 is consistent with previous reports of increased [Ca^2+^]_cyt_ in HD neurons (37, 81). Moreover, the observation that fixing RyR2-mediated ER Ca^2+^ leak with S107 improves cognitive and locomotor function in a murine model of HD (Q175 mice) suggests that leaky RyR2 play a heretofore crucial role in HD pathogenesis. This result is consistent with the in vitro neuroprotection and improved motor function previously reported using dantrolene in YAC128 mice (66, 80). Indeed, increased cytosolic Ca^2+^ concentrations can activate Ca^2+^-dependent kinases, such as CaMKII, which may contribute to further RyR2 remodeling and Ca^2+^ leak (82). Activation of CaMKII has been reported in cardiac tissues, cortex, and striatum of BACHD and R6/2 murine models of HD, where the expression of CaMKIV was increased (83, 84). Of note, HD likely shares common features with AD, in which enhanced RyR-mediated ER Ca^2+^ leak has been linked to pathological posttranslational modifications (e.g., PKA phosphorylation, oxidation/nitrosylation, and calstabin depletion) (58).

While the mechanisms involved in HD remain unclear, several hypotheses have been put forward: mutated huntingtin aggregates form inclusion bodies inside neurons, and insoluble huntingtin causes mitochondrial dysfunction, as well as Ca^2+^ dyshomeostasis and defective protein-protein interactions and vesicular transport of proteins including neurotransmitter receptors, ultimately leading to neuronal death. Moreover, mutated huntingtin microaggregates increase ROS production in the brain (85). Here we show increased ROS production in Q175 brains and increased RyR2 oxidation, which causes ER Ca^2+^ leak (Supplemental Figure 3A).

### Widespread pathology beyond the brain in HD.

In addition to the classic symptoms, HD is complicated by weight loss, heart failure, and respiratory and swallowing muscle dysfunction. These features can appear early in the disease course and can eventually contribute substantially to both morbidity and mortality (4). Heart failure occurs in about 30% of patients with HD and is a leading cause of death (86). Moreover, many patients with HD report respiratory symptoms at late stages of the disease, when the impaired motor control of the swallowing and respiratory muscle increases the risk of aspiration pneumonia (7, 14, 87). However, little is known about the pathophysiological mechanism underlying the cardiorespiratory dysfunction in HD.

We found significant remodeling of RyR2 in the cortex and the hippocampus, likely related to cognitive function impairment, and in brainstem areas involved in autonomic regulation of cardiac activity (88). RyR2 remodeling includes PKA hyperphosphorylation, oxidation, nitrosylation, and calstabin depletion. These changes represent the biochemical signature of leaky RyR2. ER Ca^2+^ leak may contribute to neurodegeneration (54) and could explain in part the dysregulation of the ANS resulting in decreased HRV, which has been observed in patients with HD and in murine HD models (25, 27, 88). Signaling from the central autonomic network is mediated through the preganglionic sympathetic and parasympathetic neurons. The most widespread cranial parasympathetic output is carried through the vagus nerve, which originates from the DMNV and NA and regulates cardiac activity. The increase in HR in Q175 mice is consistent with reduced parasympathetic activity. Our observations are consistent with the reduced HRV in patients with HD (89), including presymptomatic HD mutation carriers and mildly disabled HD patients (90). Further evidence of sympathovagal dysfunction includes symptoms such as orthostatic dizziness and tachycardia (25).

It has been suggested that sympathovagal dysautonomia in favor of sympathetic drive could result in fatal cardiac arrhythmias and/or promote heart failure in HD (28). Indeed, augmented sympathetic outflow and decreased vagal activity are considered proarrhythmic (91).

A complex network of neurons present mainly in the ventrolateral medulla oblongata and dorsolateral pons forms the respiratory central pattern generator (92). We found an overall brainstem remodeling of RyR2 and decreased diaphragmatic force production measured ex vivo in Q175 mice. In addition respiratory function was impaired at 10 months of age, manifested as a decreased TV and MV response to hypercapnia, indicative of a central respiratory defect. Interestingly, in a murine model of Duchenne muscular dystrophy, *mdx* mice, there is normal breathing at rest despite diaphragmatic weakness (93, 94). Our results are in accordance with previous data obtained in patients with HD, where respiratory pressure, forced vital capacity, peak expiratory flow, and maximum voluntary ventilation were reduced compared with controls (13). HD symptoms have been suggested to be due to neurological dysfunction or secondary to a systemic illness due to mutated huntingtin protein expressed in the peripheral tissues (2, 95, 96). To our knowledge, this is the first study evaluating the effects of huntingtin mutation in the brain, the heart, the diaphragm, and central versus peripheral pathophysiological mechanisms involved in HD. Because only S107 but not ARM036 prevented cardiorespiratory dysfunction, we conclude that leaky RyRs in the brainstem play an important role in HD pathology.

## Methods

### Animals.

For the animal model, 10-month-old Q175-Z heterozygous (human mutant Htt [mHtt] allele with the expanded CAG repeat approximately 179 repeats within the native mouse huntingtin gene) mice and age-matched WT littermates and R6/2 (contains N-terminally truncated mHtt with CAG repeat expansion [~125 repeats] within the HTT gene exon 1) were purchased from The Jackson Laboratory. Both male and female mice were used in this study. All in vivo animal experiments were performed by investigators blinded to genotype and treatment groups.

### S107 and ARM036 treatment.

The Rycal S107 (BBB permeant) was administered in drinking water at 75 mg/kg/d for 1 month as previously described (54). In order to differentiate between CNS effects versus peripheral muscle effects, the Rycal ARM036 (BBB nonpermeant) was administered in drinking water at 20 mg/kg/d. Standard food was provided ad libitum throughout the experiments. Mouse weights and brain drug levels were as described in Supplemental Figure 3, B and C.

### Immunoprecipitation.

RyR1 and RyR2 were immunoprecipitated from the diaphragm, heart, and brain using an anti-RyR1 or anti-RyR2 antibody (2 μg) in 0.5 mL of a modified RIPA buffer (50 mM Tris-HCl at pH 7.2, 0.9% NaCl, 5.0 mM NaF, 1.0 mM Na_3_VO_4_, 1% Triton X-100, and protease inhibitors) overnight at 4°C. The RyR1-specific antibody was RyR1-1327, an affinity-purified rabbit polyclonal antibody raised against a keyhole limpet hemocyanin–conjugated peptide with the amino acid sequence CAEPDTDYENLRRS, corresponding to residues 1327–1339 of mouse skeletal RyR1, with an additional cysteine residue added to the amino terminus, and affinity purified with the unconjugated peptide (78). The RyR2-specific antibody was an affinity-purified polyclonal rabbit antibody using the peptide CKPEFNNHKDYAQEK corresponding to amino acids 1367–1380 of mouse RyR2 with a cysteine residue added to the amino terminus (60). The immune complexes were incubated with protein A–Sepharose beads (MilliporeSigma) at 4°C for 1 hour, and the beads were washed 3 times with RIPA buffer. The immunoprecipitates were size fractionated on SDS-PAGE gels (4%–20% for RyR1/RyR2 and calstabin) and transferred onto nitrocellulose membranes for 2 hours at 200 mA. Immunoblots were developed using the following primary antibodies: anti-RyR1/2 (Affinity BioReagents, 1:2000, ab55999), anti–phospho-RyR-Ser(P)-2808 (Affinity BioReagents, 1:5000, ab59225), anti-calstabin (FKBP12 C-19, SC-133067, 1:1000, Santa Cruz Biotechnology, Inc.), and anti–Cys-NO (Y061263, 1:1000, MilliporeSigma). To determine channel oxidation, the carbonyl groups in the protein side chains were derivatized to DNP by reaction with 2,4-dinitrophenylhydrazine. The DNP signal associated with RyR was determined using a specific anti-DNP antibody according to the manufacturer’s instructions (MilliporeSigma S7150). All immunoblots were developed using an Odyssey system (LI-COR Biosciences) with infrared-labeled anti–mouse and anti–rabbit IgG (1:10,000, 926-32211) secondary antibodies.

### SR vesicle preparation.

Brains, hearts, and diaphragms were homogenized on ice in 300 mM sucrose, with 20 mM 1,4-piperazinediethanesulfonic acid (PIPES, MilliporeSigma) (pH 7.0) in the presence of protease inhibitors (Roche), and centrifuged at 5900*g* for 20 minutes at 4°C. The supernatant was ultracentrifuged at 100,000*g* for 1 hour at 4°C. The final pellet containing microsomal fractions enriched in SR vesicles was resuspended and aliquoted in 300 mM sucrose, with 5 mM PIPES (pH 7.0) containing protease inhibitors. Samples were frozen in liquid nitrogen and stored at −80°C.

### Single-channel data using planar lipid bilayers.

Planar lipid bilayers were formed using a 3:1 mixture of phosphatidylethanolamine and phosphatidylcholine (Avanti Polar Lipids) suspended (30 mg/mL) in decane by painting the lipid/decane solution across a 200 μm aperture in a polysulfonate cup (Warner Instruments) separating 2 chambers. The trans chamber (1 mL) representing the intra-ER/SR (luminal) compartment was connected to the headstage input of a bilayer voltage clamp amplifier (BC-525D, Warner Instruments), and the cis chamber (1 mL), representing the cytoplasmic compartment, was held at virtual ground. Solutions in both chambers were as follows: 1 mM EGTA, 250/125 mM HEPES/Tris, 50 mM KCl, and 0.64 mM CaCl_2_, pH 7.35 as cis solution and 53 mM Ca(OH)_2_, 50 mM KCl, 2and 50 mM HEPES, pH 7.35 as trans solution. The concentration of free Ca^2+^ in the cis chamber was calculated using the WinMaxC program (version 2.50). SR vesicles were added to the cis side, and fusion with the lipid bilayer was induced by making the cis side hyperosmotic by the addition of 400–500 mM KCl. After the appearance of potassium and chloride channels, the cis compartment was perfused with the cis solution. Single-channel currents were recorded at 0 mV by using a Bilayer Clamp BC-535 amplifier (Warner Instruments), filtered at 1 kHz, and digitized at 4 kHz. All experiments were performed at room temperature. Data acquisition was performed using Digidata 1440A and Axoscope 10.2 software; recordings were analyzed using Clampfit 10.2 (Molecular Devices, Thermo Fisher Scientific). P_O_ was identified by 50% threshold analyses using a minimum of 2 minutes of continuous record. At the conclusion of each experiment, ryanodine (5 μM) was added to the cis chamber to confirm channels as RyR.

### Brain immunohistochemistry.

Mice were perfused transcardially with 30 mL phosphate-buffered saline followed by 30 mL of 4% formaldehyde. Brainstems were removed and postfixed for 12 hours in 4% formaldehyde at 4°C. All brainstems were cut coronally (50 μm sections) using a vibratome. Fluorescence immunohistochemistry was performed as previously described (97, 98). Sections were immunostained for detection of TH, ChAT, and either RyR2 or P*RyR2. The primary antibodies used were mouse anti-TH (1:500, Merck, MAB318), goat anti-ChAT (1:200, Merck, AB144P), rabbit anti-RyR2 (60) (1:400), and rabbit anti-P*RyR2 (1:400, ab59225, Affinity BioReagents). The secondary antibodies used were Alexa Fluor 647 donkey anti-mouse (1:500, Invitrogen, Thermo Fisher Scientific, A31571), Alexa Fluor 555 donkey anti-rabbit (1:500, Invitrogen, Thermo Fisher Scientific, A31572), and Alexa Fluor 488 donkey anti-goat (1:500, Invitrogen, Thermo Fisher Scientific, A11055). The presence of RyR2 or P*RyR2 immunoreactivity in presympathetic adrenergic C1 neurons of the rostral ventrolateral medulla oblongata (TH immunoreactive) and cardiac parasympathetic preganglionic neurons of the NA (ChAT immunoreactive) was examined using a ZEISS Axioplan 2 microscope with an Apotome module. Brainstem sections were sampled every 100 μm, and bilateral images (×20 objective) of typically 2–3 sections per animal were acquired using 9 tiles of *Z*-stacks composed of 8 optical sections (8 μm focal spacing). For Figure 3A, a maximum intensity projection of optical sections was performed. Semiquantitative analyses of colocalization between RyR2 or P*RyR2 immunoreactivity and TH or ChAT immunoreactivity were performed in 4 WT and 4 Q175 mice, using the following code: - = 0 colocalized neurons; + = 1%–25% colocalized neurons; ++ = 26%–50% colocalized neurons; +++ = 51%–75% colocalized neurons; and ++++ = 76%–100% colocalized neurons.

### Measurement of diaphragm fibers’ cross-sectional areas.

Frozen diaphragm muscle strips from the left middle segment of costal diaphragms were embedded in OCT for cryosections. Cryosections (10 μm) were taken precisely perpendicular to the fibers by a previously described technique (99), fixed with cold isopentane for 2 minutes, and immunostained with murine anti–α-myosin heavy chain (MilliporeSigma, 1:2000, M7421). Muscle membrane was counterstained with rabbit anti-dystrophin antibodies (Abcam, 1:1000, ab230379) using standard procedures. Alexa Fluor 555 anti-mouse and Alexa Fluor 488 anti-rabbit secondary antibody (Thermo Fisher Scientific A11094 and A32727) were then applied. Slides were mounted in ProLong Gold antifading reagent (Invitrogen, Thermo Fisher Scientific) and imaged by confocal fluorescence microscopy. The cross-sectional area (CSA) was measured using Fiji (http://fiji.sc/wiki/index.php/Fiji) software, an enhanced version of ImageJ (NIH). Two regions from each section (total ~150 fibers/sample) were processed.

### Contractile function in murine muscle samples.

Mice were euthanized by exsanguination and the entire diaphragm EDL or soleus was surgically excised. Isometric contractile properties were assessed as described elsewhere (100). The excised diaphragm strip, EDL, and soleus were mounted into jacketed tissue bath chambers filled with equilibrated and oxygenated Krebs solution. The muscles were supramaximally stimulated using square wave pulses (Model S48; Grass Instruments). The force-frequency relationship was determined by sequentially stimulating the muscles for 600 ms at 10, 20, 30, 50, 60, 80, 100, and 120 Hz with 1 minute between each stimulation train (53). After measurement of contractile properties, muscles were measured at the length at which the muscle produced maximal isometric tension, dried, and weighed. For comparative purposes, muscle force production was normalized for total muscle strip CSA and expressed in N/cm^2^. The total muscle strip CSA was determined by dividing muscle weight by its length and tissue density (1.056 g/cm^3^).

### Behavioral studies.

EPM and MWM tests were performed as previously described (54, 58). Novel object recognition test was performed as previously described (101). Briefly, the EPM test was performed using the EPM apparatus, which consisted of 4 arms (2 open without walls and 2 enclosed by 15 cm high walls), 68 cm long and 5 cm wide, was elevated 55 cm off the floor. A video tracking system (Noldus Information Technology Inc.) with computer interface and video camera were used to automatically collect behavioral data. Each mouse was placed at the junction of the 4 arms of the maze; numbers of entries and the time spent in each arm were recorded by the examiner and the video tracking system simultaneously for 5 minutes. Deodar wiper was used between each tested mouse. The ratio of total time spent in, and the numbers of entry to the open arm versus closed arm, of each mouse were analyzed.

Spatial learning and memory function were evaluated using MWM (102) task, which consisted of a circular pool (122 cm in diameter, 76 cm in depth) (San Diego Instruments) in which mice were trained to escape from water by swimming to a 10 cm diameter hidden platform (1.0 to 1.5 cm underneath water surface). Water temperature was maintained at 23°C ± 1°C, and the water was rendered opaque by the addition of white nontoxic paint (Discount School Supply). The pool was divided into 4 quadrants by a computerized tracking/image analyzing system (Noldus Information Technology Inc.). The hidden platform was placed in the middle of the northwest (NW) quadrant and remained in the same position during the experiment. The spatial acquisition phase consisted of 15 training trials: 5 training days and 3 trials per day with an intertrial interval of 40–60 minutes. Mice were released from different quadrants between trials with their heads facing the pool wall of 1 of the 4 compass locations (SW, SE, and NE) and allowed to swim and search for the platform for 60 seconds. The latency to reach the hidden platform, travel path, and swimming velocity were recorded by the examiner and the video tracking system simultaneously. On day 6, memory retention was evaluated by a probe trial 24 hours after the last training session in the absence of the escape platform. Mice were allowed to swim freely for 60 seconds. The location where the hidden platform was previously located was defined as the target. The northwest quadrant where the platform was previously hidden was defined as the target quadrant. The number of target crossings and the proportion of swimming time spent in the target quadrant were recorded and analyzed.

The tail suspension test was used to assess depression-related behavior, as described previously (102). Briefly, on the top of a box, a suspension bar was used to suspend the tail of the mouse. Each mouse was suspended separately in the middle of the box, using black adhesive tape applied to the end of the tail (with 2–3 mm remaining outside the tape), and the free end of the tape was attached to the middle of the suspension bar. The approximate distance between the mouse’s nose and the apparatus floor was 10–15 cm. Every session lasted for 6 minutes and was recorded using a video camera placed on a tripod in front of the box. The time that each mouse spent immobile was measured. Lack of escape-related behavior is considered immobility, which is indicative of depression-like behavior.

### Whole-body plethysmography measurement.

Respiratory function was measured in conscious, unrestrained mice using whole-body plethysmography and analyzed using iox2 software (EMKA Technologies) as previously described (94, 103). Mice were randomly placed into individual chambers. After 1-hour stabilization, each animal was visually monitored, and ventilatory parameters were recorded during an inactive phase for 5 minutes under 2 experimental conditions: first, room air breathing, then under hypercapnic conditions using 8% CO_2_-enriched airflow to activate respiration. Air volume changes corresponding to spontaneous breathing were obtained using a pressure transducer (EMKA Technologies). This pressure signal was used to calculate ventilatory parameters: TV, respiratory rate, MV, inspiratory time, and expiratory time.

### Running wheel.

Mice were subjected to voluntary aerobic exercise with free access to a running wheel (diameter 11.5 cm), which was connected to a digital counter for 24 hours (light/dark cycle), as previously described (104).

### ECG recording in conscious animals.

Mice were implanted with radio telemetry transmitters (Data Sciences International) as described in detail elsewhere (105). Briefly, the transmitter (PhysioTel, ETA-F10 transmitter) was inserted in mice subcutaneously along the back under general anesthesia (20% inhaled isoflurane/O_2_, Aerrane) coupled with local anesthetic (lidocaine 0.5%), and 2 ECG electrodes were placed hypodermically in the region of the right shoulder (negative pole) and toward the lower left chest (positive pole) to approximate lead II of the Einthoven surface ECG. During the procedure, respiratory and cardiac rhythm, adequacy of anesthetic depth, muscle relaxation, body temperature, and analgesia were monitored to avoid anesthesia-related complications. Postoperating pain was considered during a 1-week postimplantation period and buprenorphine (0.3 mg/kg s.c.) was given. A minimum period of 2 weeks was allowed for recovery from the surgery. Animals were housed in individual stainless steel cages for telemetry recordings. Environmental parameters were recorded continuously and maintained within a fixed range: room temperature at 15°C–21°C and 45%–65% relative humidity. The artificial day/night cycle was 12-hour light/12-hour dark with light on at 0700 hours. Drinking water was provided ad libitum. Solid diet (300 g) was given daily in the morning. ECG waveforms were continuously recorded at a sampling rate of 2000 Hz using a signal transmitter-receiver (RPC-1) connected to a data acquisition system (Ponemah system, Data Sciences International). Continuous digital recordings were analyzed offline after being digitally filtered between 0.1 and 1000 Hz. ECGs during nocturnal and diurnal periods (12-hour light/12-hour dark cycle) were analyzed with Ponemah software using template automatic detection, secondly validated by an operator. The mean RR interval and QT durations were calculated. The QT interval was defined as the time between the first deviation from an isoelectric PR interval until the return of the ventricular repolarization to the isoelectric TP baseline from lead II ECGs.

Presence of ectopic beats was scanned by hand. Then, HRV was evaluated by power spectral analysis (ms^2^) using the fast Fourier transformation (segment length of 2048 beats, linear interpolation with resampling to a 20 Hz interbeat time series and Hamming windowing). The cutoff frequency ranges for the LFr powers (HFr: 1.5–5 Hz) were chosen according to those used in the literature (106).

### Autonomic blockade.

Complete suppression of autonomic control was achieved with ganglioplegic compound hexamethonium (20 mg/kg, i.p.).

Further information can be found in Supplemental Methods.

### Statistics.

Group data are presented as mean ± SD unless otherwise indicated. Statistical comparisons between 2 groups were tested using an unpaired 2-tailed *t* test. ANOVA tests (1 and 2 way) with a Bonferroni’s post hoc adjustment were used for multiple comparisons. Values of *P* < 0.05 were considered statistically significant. All statistical analyses were performed with GraphPad Prism 8.0.

### Study approval.

For human samples, deidentified human hippocampus and cortex samples were obtained from the New York Brain Bank at Columbia University. The age and sex of these samples were 64, female; 58, female; 54, female; 61, female; 66, female; 56, male; 58, male; and 63, male. Age- and sex-matched controls exhibited absence of neurological disorders and plaques, and previous experiments using these specific control samples had shown a lack of remodeling and leak in RyR2 (54). Information on the patients with HD is listed in Supplemental Table 1.

For the animal model, all mice were maintained and studied according to protocols approved by the Institutional Animal Care and Use Committee of Columbia University (reference AC-AAAV5455) and Directive 2010/63/EU of the European parliament and the Council of September 22, 2010, for the protection of animals used for scientific purposes and ethics committee for animals experiments, Languedoc Roussillon, C2EA-36 (agreement B34-172-38; project APAFIS 13528-2018). Q111 frozen brain tissues were obtained from the CHDI Foundation (New York, New York, USA) for only RyR2 analysis. We chose 10-month-old Q175 mice when the mice exhibited remarkable behavioral deficits accompanied by marked brain atrophy and brain metabolite changes and started developing respiratory and autonomic dysfunction (107).

## Author contributions

HD, AL, and ARM designed experiments, analyzed data, and edited or wrote the paper. XL, QY, SR, MY, LS, PA, JT, PS, SM, CM, and JB designed experiments and analyzed data.

## Supplementary Material

Supplemental data

## Version 1 09/08/2020

In-Press Preview

## Version 2 10/02/2020

Electronic publication

## Version 3 10/02/2020

The spelling of Mohamad Yehya's name was corrected.
