# Supplementary material for: Role of defective calcium regulation in cardiorespiratory dysfunction in Huntington’s disease
Source: JCI Insight. 2020 Oct 2;5(19):e140614. doi: 10.1172/jci.insight.140614 (PMC7566717; doi:10.1172/jci.insight.140614)
Supplement: Supplemental data [file jciinsight-5-140614-s259.pdf]

Age (years) /Sex	Post-mortem Interval (hours)	Neuropathological Grade	CAG Repeat Length
64, female	18	4	36
58, female	22	4	50
54, female	16	4	40
61, female	20	3	36
66, female	18	4	41
56, male	20	3	38
58, male	16	4	51
63, male	18	4	42
85, female	within 24 hours	-	Control
84, female	within 24 hours	-	Control
92, female	within 24 hours	-	Control
81, male	within 24 hours	-	Control

Table 1: demographic data and neuropathological findings related to human samples. Hippocampus and cortex were harvested from HD patients (54-66 years old) within 16-22 hours post-mortem. These patients were with severe neuropathological grade (3-4) (see Neuropathological classification of Huntington's disease[1]). CAG repeats were between 36 and 51. Controls were between 84-92 years old and were negative for any neuropathological diagnoses. Hippocampus and cortex were harvested with 24 hours post-mortem

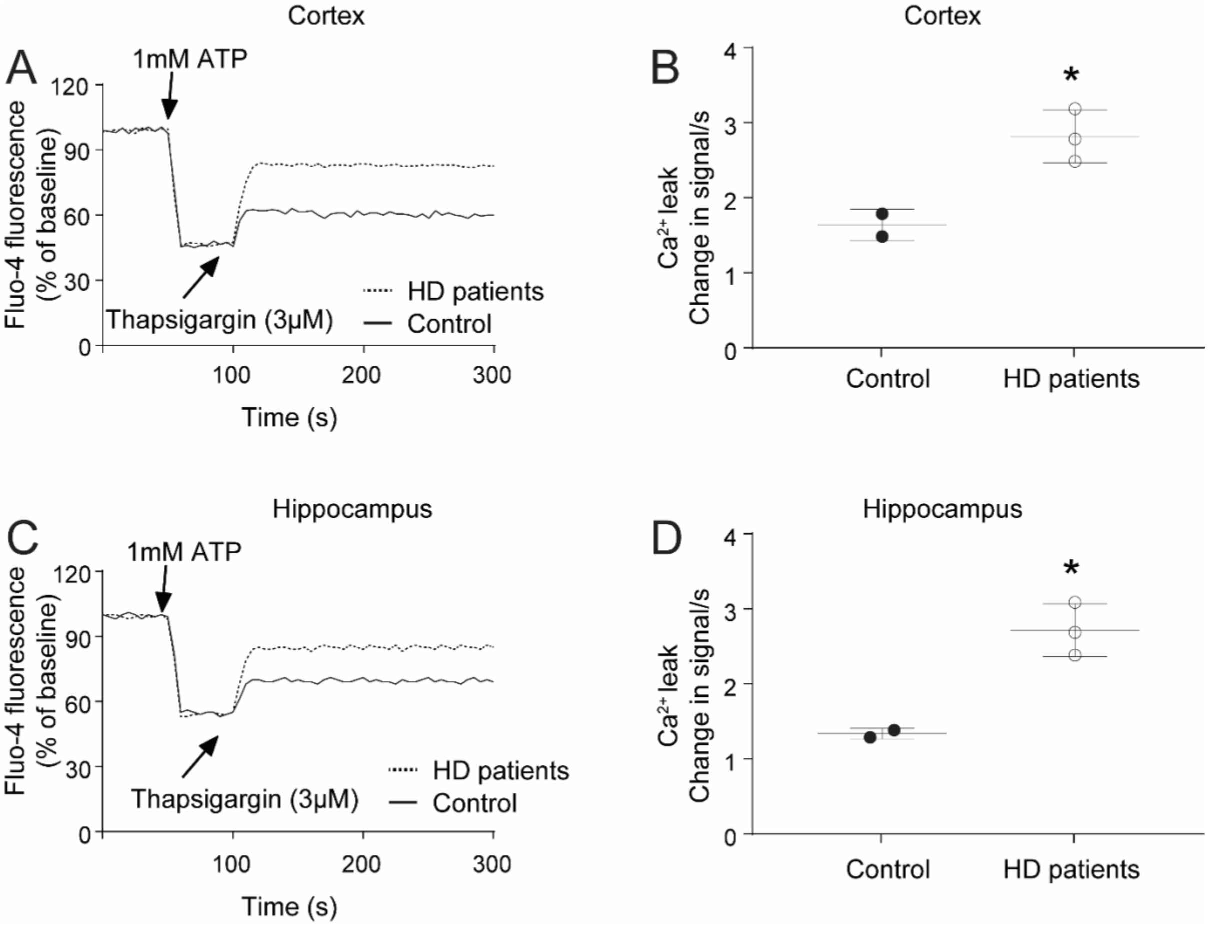

Figure S1: Endoplasmic reticulum (ER) Ca^{2+} leak in cortical and hippocampal neurons of HD patients and Control. A and **C)** Representative traces of Ca^{2+} leak, from cortex and Hippocampus microsomes from control (n=2) and HD patients (n=3), induced by addition of thapsigargin (3µM). Endoplasmic reticulum Ca^{2+} loading was initiated by adding 1mM of ATP. **(B** and **D)** Ca^{2+} leak was calculated as the change in fluorescence signal per second. Data (mean ± SD) analysis was performed by t-test * $p<0.05$ vs. Control.

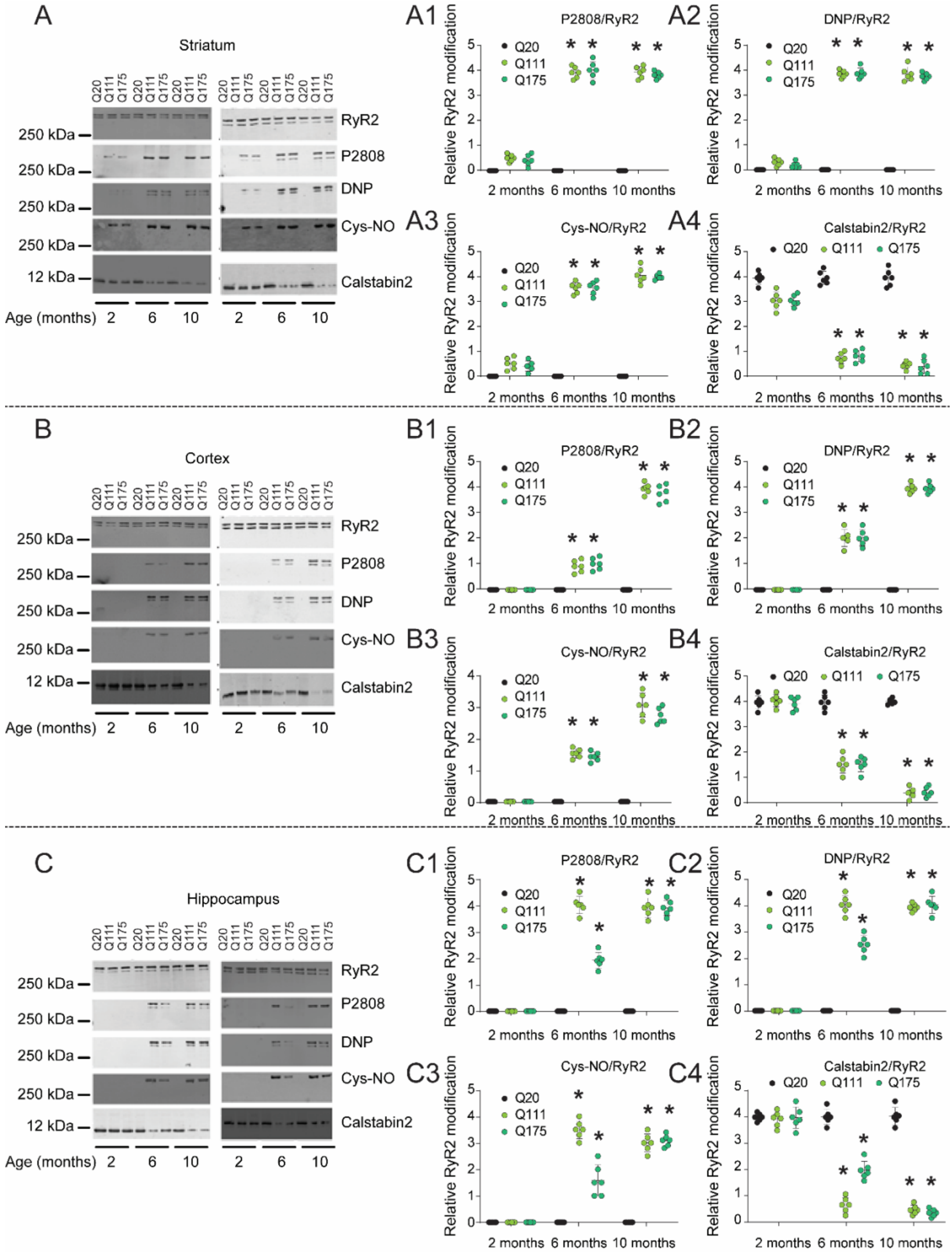
A
Striatum
Q20
Q111
Q175
RyR2
P2808
DNP
Cys-NO
Calstabin2
250 kDa
12 kDa
Age (months) 2 6 10 2 6 10
A1
P2808/RyR2
A2
DNP/RyR2
A3
Cys-NO/RyR2
A4
Calstabin2/RyR2
Relative RyR2 modification
2 months 6 months 10 months
B
Cortex
B1
P2808/RyR2
B2
DNP/RyR2
B3
Cys-NO/RyR2
B4
Calstabin2/RyR2
C
Hippocampus
C1
P2808/RyR2
C2
DNP/RyR2
C3
Cys-NO/RyR2
C4
Calstabin2/RyR2

Figure S2: Age-related striatum RyR2 remodeling in 2 HD mouse model (Q111 and Q175) in striatal, cortical and hippocampal neurons. Top panel: RyR2 immunoprecipitation from striatum lysates from control (Q20) and HD (Q111. Q175) mice. RyR2 was immunoprecipitated from striatum lysates. **(A)** show 2 representative set of immunoblots comparing RyR2 phosphorylation, oxidation, nitrosylation, and calstabin2/RyR2 association in control lysates versus lysates from **Q111** and **Q175** mice at 2, 6 and 10 months. Bar graphs depict quantification of the relative phosphorylation of RyR2 **(A1)**, oxidation of RyR2 **(A2)**, nitrosylation **(A3)**, and Calstabin2 **(4)** bound to the channel (n = 6 for each group). Middle panel: RyR2 immunoprecipitation from cortex lysates from control (Q20) and HD (Q111. Q175) mice. RyR2 was immunoprecipitated from cortex lysates. **(B)** show 2 representative set of immunoblots comparing RyR2 phosphorylation, oxidation, nitrosylation, and calstabin2/RyR2 association in control lysates versus lysates from Q111 and Q175 mice at 2, 6 and 10 months. Bar graphs depict quantification RyR2 modifications in the cortex. Phosphorylation of RyR2 **(B1)**, oxidation of RyR2 **(B2)**, nitrosylation **(B3)**, and Calstabin2 **(B4)** bound to the channel (n = 6 for each group). Bottom panel: RyR2 immunoprecipitation from hippocampus lysates from control (Q20) and HD (Q111. Q175) mice. RyR2 was immunoprecipitated from hippocampus lysates. **(C)** show 2 representative set of immunoblots comparing RyR2 phosphorylation, oxidation, nitrosylation, and calstabin2/RyR2 association in control lysates versus lysates from Q111 and Q175 mice at 2, 6 and 10 months. Bar graphs depict quantification RyR2 modifications in the hippocampus. Phosphorylation of RyR2 **(C1)**, oxidation of RyR2 **(C2)**, nitrosylation **(C3)**, and Calstabin2 **(C4)** bound to the channel (n = 6 for each group). Data (mean ± SD) analysis was performed by one-way ANOVA. Bonferroni post-test revealed * p<0.05 vs. WT.

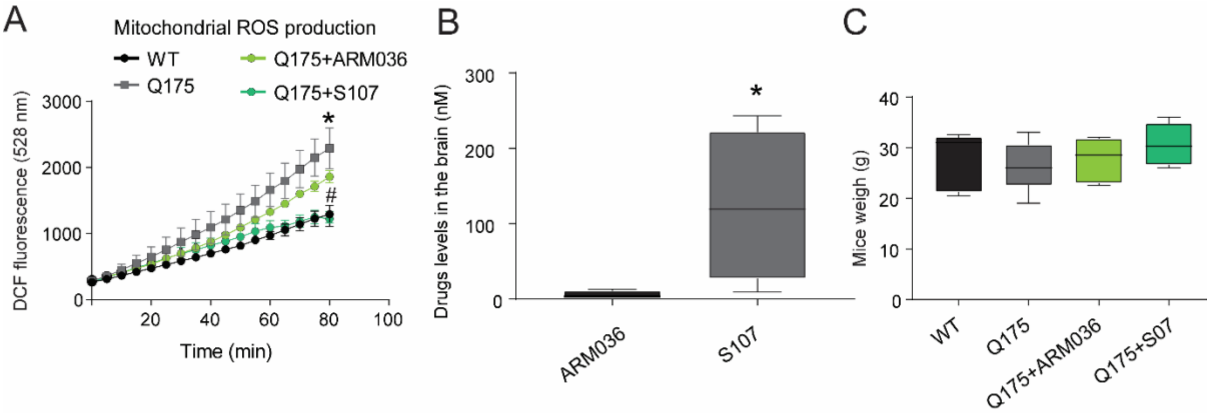

Figure S3: Mitochondrial ROS production in whole brain from HD mice samples, brain drugs level and mice weight. A) Mitochondrial reactive oxygen species (ROS) production were measured using DCF-DA method. DCF fluorescence was measured at 490/528 nm during 80 min using a multimode plate reader. DCF fluorescence in WT, Q175, Q175+S107 and Q175+ARM036 group (n=4 in each group) recorded from whole brain samples during 80 minutes. Data (mean ± SEM) analysis was performed by Two-way ANOVA. Bonferroni post-test revealed * p<0.05 vs. WT, # p<0.05 vs. Q175. **B)** ARM036 and S107 brain levels concentration in nanomolar measured by Mass spectrometry. Quantified data are represented as a box-and-whisker plot, with bonds from 25th to 75th percentile, median line, and whiskers ranging from minimum to maximum values. Student t-test *p<0.05 ARM036 vs S107. **C)** Mice weight in all experimental groups. Data are presented as a box-and-whisker plot, with bonds from 25th to 75th percentile, median line, and whiskers ranging from minimum to maximum values.

Test	WT	Q175	Q175+S107
**Ledge (0-3)**	0	1.75±0.25*	0.33±0.21#
**Hind limb clasping (0-3)**	0	1.38±0.26*	0.67±0.21#
**Gait (0-3)**	0	0.37±0.18	0
**Kyphosis (0-3)**	0	0	0

Table 2: Motor neuron function in Q175 mice. Ledge, hind limb clasping, gait and kyphosis in WT (n=8), Q175 (n=6) and Q175 treated mice with S107 (n=6) at 10 months of age. Data (mean ± SD) analysis was performed by One-way ANOVA. Bonferroni post-test revealed * $p<0.05$ vs. WT, # $p<0.05$ vs. Q175.

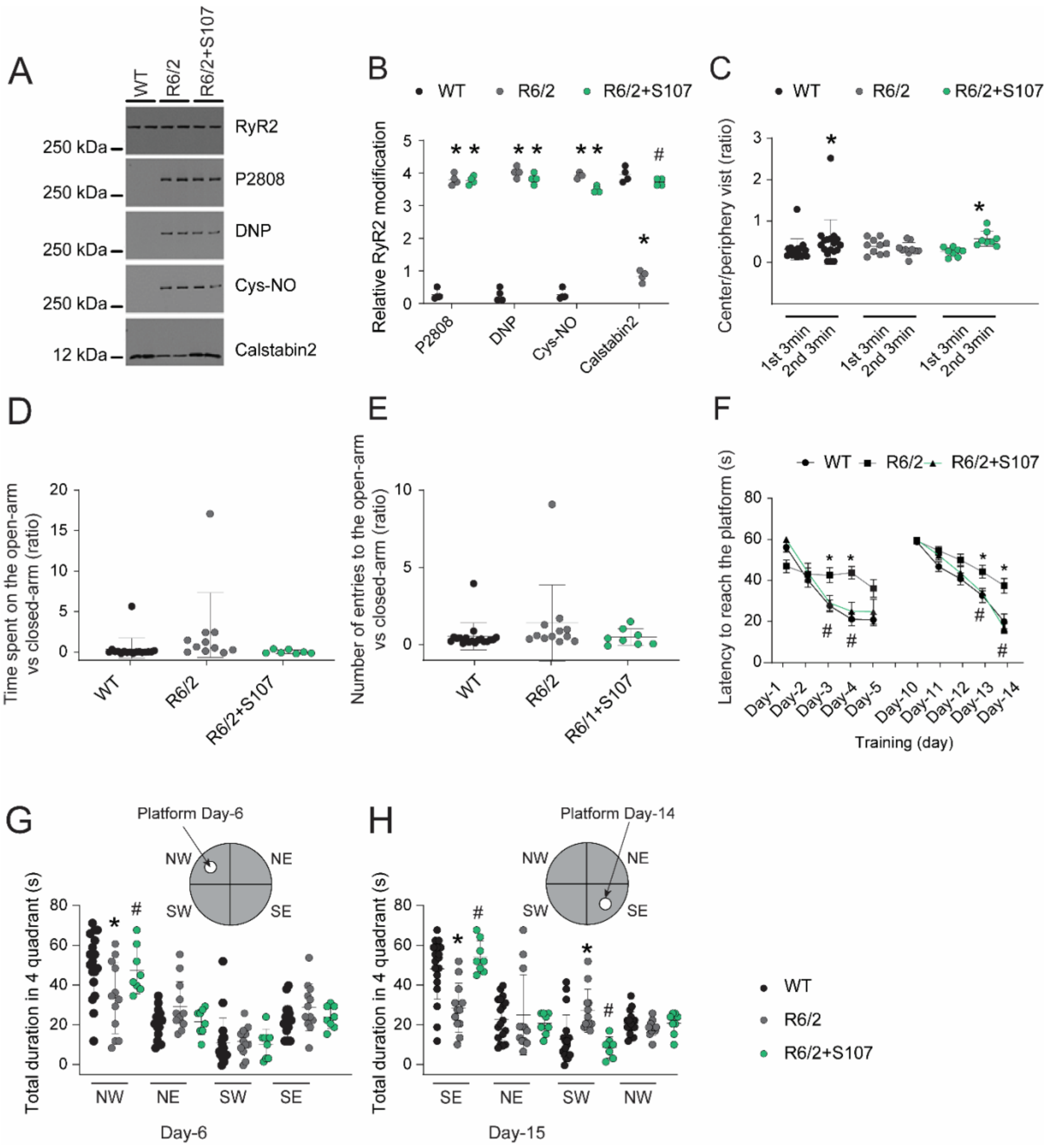

Figure S4: RyR2 Channel remodeling and Long-term learning and memory deficits in R6/1 mice. Representative SDS-PAGE analysis **(A)** and quantification of RyR2 **(B)** immunoprecipitated from whole brain lysates (band intensity were normalized to total RyR2). The blots show RyR2 phosphorylation, oxidation, nitrosylation, and calstabin2/RyR2 association in WT (n=4), R6/1 (n=4) and R6/1 mice treated with S107 (n=4). **C)** Results from open-field test are shown: the number of visits to the center area and peripheral area during the 1st 3min and 2nd 3min were recorded and analyzed separately. Results from elevated plus maze are shown. The ratio of time spent in the open-arm versus closed-arm and the ratio of the total number of entries to the open-arm versus closed-arm were recorded **(D, E)**. **F)** MWM escape latency for WT (n=18), R6/1 (n=12), and S107 treated mice R6/1+S107 (n=8) during the 5-day trials when the hidden platform was initially located in the northeast quadrant (NW) **(F-**on the left**)**. After a 3-day interval, the hidden platform was relocated to the southeast quadrant (SE) **(F** on the right**)**. Learning curves show the escape latency for the same groups

of mice to reach the relocated hidden platform during another 5-days of training **(F)** Data (mean ± SEM) analysis was performed by Two-way ANOVA. Bonferroni post-test revealed * p<0.05 vs. WT, # p<0.05 vs. R6/2. Two separate probe trials immediately followed each 5-day training trial. The time spent in the target quadrant (NW and SE) for each group is shown **(G and H)**. NE: Northeast quadrant, NW: Northwest quadrant, SE: Southeast quadrant, SW: southwest quadrant. Data (mean ± SD) analysis was performed by One-way ANOVA. Bonferroni post-test revealed * p<0.05 vs. WT, # p<0.05 vs. R6/2.

**Age (month)**	**Group**	**Gait**	**Kyphosis**	**Clasping**	**Ledge**
**3-4**	WT	0	0	0	0
	R6/2	0	0	1.79±1.19*	1.86±1.1*
	R6/2+S107	0	0	0	0.89±0.3#
**6-7**	WT	0	0	0	0
	R6/2	0	0.6±0.4*	2.4±0.4*	03±0*
	R6/2+S107	0	0	0.67±0.33	0.5±0.22#
**10**	WT	0	0	0.5±0.3	0.7±0.5
	R6/2	2.4±0.4*	1.8±0.2*	3±3*	3±0*
	R6/2+S107	1±0.4#	0.25±0.25	1.5±0.3#	1.5±0.3#

Table 3: Motor neuron function in R6/2 mice: Gait, kyphosis, clasping and ledge in WT (n=18), R6/2 (n=12) and R6/2 treated mice with S107 (n=8) at 3-4 months, 6-7 months and 10 months. Data (mean ± SD) analysis was performed by One-way ANOVA. Bonferroni post-test revealed * p<0.05 vs. WT, # p<0.05 vs. R6/2.

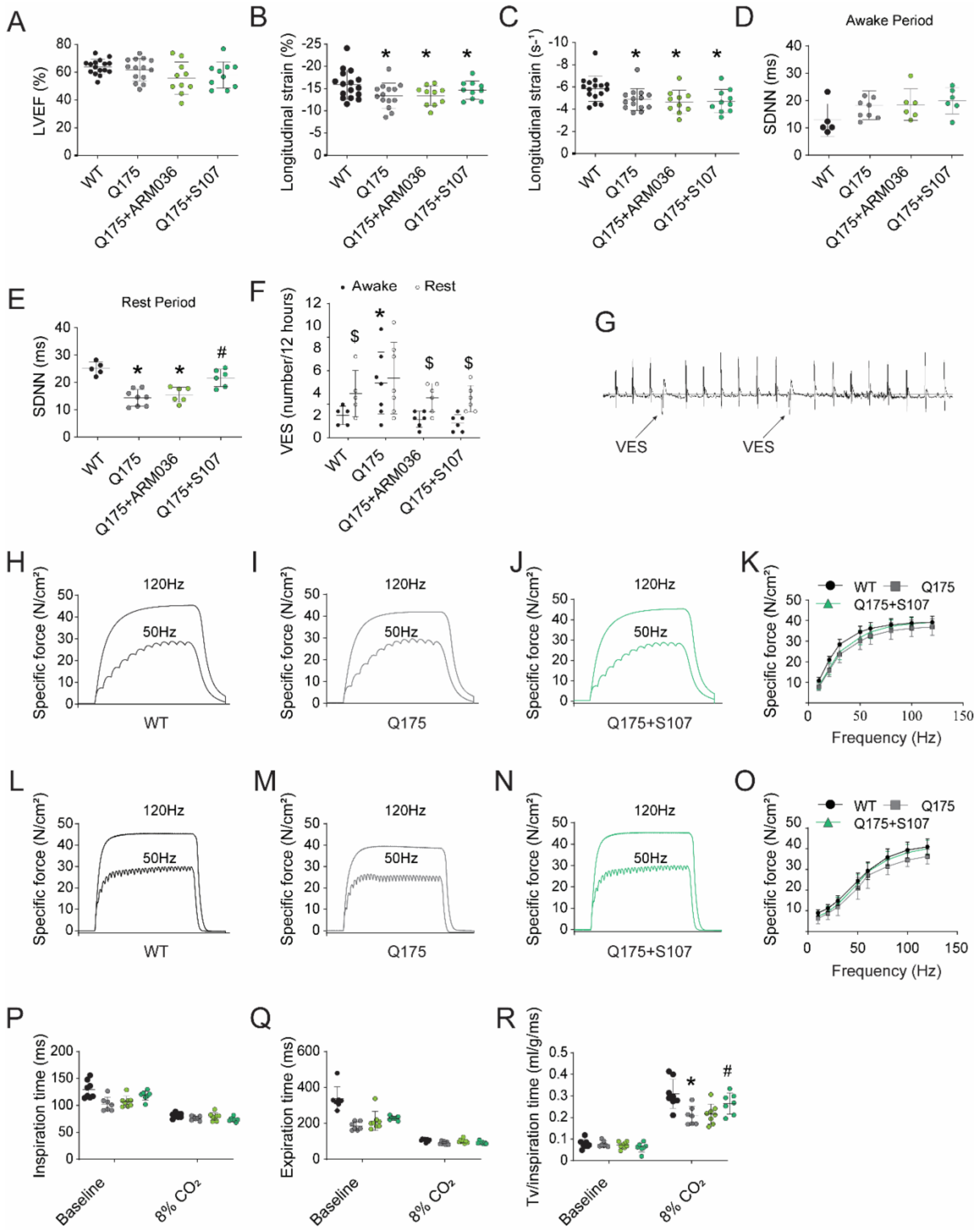

Figure S5: Heart function, ventricular extra-systoles, Ex-vivo EDL and soleus contractile function whole body respiratory function in Q175 mice. A) Ejection fraction in WT (n=16), Q175 (n=14), Q175+S107 (n=10) and Q175+ARM036 (n=10). **B)** longitudinal strain in WT (n=16), Q175 (n=14), Q175+S107 (n=10) and Q175+ARM036 (n=10). **C)** longitudinal strain

rate in WT (n=16), Q175 (n=14), Q175+S107 (n=10) and Q175+ARM036 (n=10). **D)** Heart rate variability analyzed in the time-domain (SDNN) in WT (n=5), Q175 (n=8), Q175+ARM036 (n=6) and Q175+S107 (n=6) mice during awake period. **E)** Heart rate variability analyzed in the time-domain (SDNN) in WT (n=5), Q175 (n=8), Q175+ARM036 (n=6) and Q175+S107 (n=6) mice during rest period. **F)** Number of isolated triplet (three consecutive) ventricular extra-systoles (VES) during 10 hours in WT (n=5), Q175 (n=7), Q175+ARM036 (n=6) and Q175+S107 (n=6) during rest and awake period. Data (mean ± SD) analysis was performed by One-way ANOVA. Bonferroni post-test revealed * $p<0.05$ vs. WT, # $p<0.05$ vs Q175, $ $p<0.05$ awake vs rest. **G)** Representative trace of ventricular extra-systoles (VES) in Q175 mice during rest period. **H, I and J)** Representative records of specific force production measured ex-vivo at 20 and 120 Hz in soleus muscle under isometric conditions in WT, Q175, and Q175+S107 treated mice. **K)** Average force-frequency relationship recorded in soleus from WT (n=8), Q175 (n=8), and Q175+S107 (n=8) treated mice. **L**, **M and N)** Representative records of specific force production measured *ex-vivo* at 20 and 120 Hz in EDL muscle under isometric conditions in WT, Q175, and Q175+S107 treated mice. **O)** Average force-frequency relationship recorded in EDL WT (n=8), Q175 (n=8), and Q175+S107 (n=8) treated mice. Data are mean ± SEM. **P)** Inspiratory time (msec) at rest and after CO_2 stimulation. **Q)** Expiratory time (msec) at rest and after CO_2 stimulation. **R)** Tidal volume reported to inspiratory time (ml/g/ms) at rest and after CO_2 stimulation WT (n=16), Q175 (n=14), Q175+S107 (n=10) and Q175+ARM036 (n=10). Data (mean ± SD) analysis was performed by One-way ANOVA. Bonferroni post-test revealed * $p<0.05$ vs. WT, # $p<0.05$ vs. Q175.

Supplementary Method:

Microsomal calcium leak assay: The ER Ca^{2+} leak assay was measured as previously described [2]. Briefly, 50 µg/ml diaphragm microsomes was diluted into a buffer (pH 7.2) containing 8 mM K-phosphocreatine and 2 units/ml of creatine kinase mixed with 3 µM Fluo-3. Ca^{2+} loading of the microsomes was initiated by adding 1 mM ATP. After Ca^{2+} uptake to saturation, 3 µM thapsigargin was added to block Ca^{2+} uptake. Ca^{2+} leaking out of the ER was detected as an increase in Ca^{2+} fluorescence, and ryanodine (10 µM) was added to block the RyR channels and confirm that the leak was via RyR. Changes in the emission of Fluo-4 after excitation at 485 nm were measured by a fluorescence spectrophotometer (Photon Technology International, PTI) and the changes of fluorescence intensity were recorded using "FeliX version 2" software (PTI).

Cardiac function assessment:

Standard Echocardiography: Cardiac function was assessed in anesthetized mice (1-3% isoflurane, 100% oxygen) by transthoracic echocardiography using a high-resolution ultrasound system (Vevo 2100; VisualSonics, Toronto, Canada) equipped with a 40-MHz linear array transducer (MS550, Vevo2100, VisualSonics). Animals were placed on a heating table in a supine position. Body temperature was monitored through a rectal thermometer to be maintained at 37°C and ECG was recorded all along the echocardiographic procedure with limb electrodes to ensure physiological heart rate (> 350 bpm). Left ventricular (LV) parasternal long axis 2D view in M-mode was performed at the level of papillary muscle to assess LV wall thicknesses and internal diameters, allowing the calculation of the fractional shortening (FS) and ejection fraction (EF) by the Teicholz method.

Speckle Tracking Echocardiography analysis. Global and regional left ventricular dynamics were assessed by speckle tracking analysis from B-mode cine loops acquired during the echocardiographic sessions. Parasternal long axis views were used for the evaluation of LV

longitudinal strain. Strain analysis was conducted by the same trained blinded investigator as previously described [3].

ROS production: Mice brains were placed in isolation medium (containing 225 mM mannitol, 75 mM sucrose, 5 mM HEPES, 1 mM EGTA, pH 7.4) and homogenized using a glass-teflon homogenizer. The tissue homogenate was centrifuged 5minx1000g and 10minx10,000g. Mitochondrial pellet was suspended in isolation medium and total mitochondrial protein was determined using Bradford method. Mitochondrial ROS production were measured using DCF-DA method as previously described [4] and according to manufacture instructions. Mitochondria were incubated for 1hr at 37C with 20 μM H2DCF-DA (Thermo Fisher scientific) which is activated by ROS to generate a highly fluorescent 2′7′-dichlorofluorescein (DCF) molecule. DCF fluorescence was measured at 490/528 nm during 80min using a multimode plate reader.

Mass spectrometry analysis

Brain tissues collected from treated Q175 mice were extracted with 500ul MeOH/CAN/H_2 2:2:1 (vol/vol/vol). The samples were then vortexed for 30s. For protein precipitation, the samples were sonicated for 15min and incubated 1h at -20C, then centrifuged 15min at 13,000 r.p.m and 4C. The resulting supernatant was removed and evaporated to dryness in a vaccum concentrator. Dry extracts were then reconstituted in 100 ul of CAN/H_2O 1:1 (vol/vol), sonoicated 15min and centrifuged 15min at 13,000 r.p.m and 4C to remove insoluble debris. Supernatants were transferred to HPLC vials and transferred to the mass spectrometry for small molecule analysis as previously described [5].

References

1 Vonsattel, J. P. *et al.* Neuropathological classification of Huntington's disease. *J Neuropathol Exp Neurol* **44**, 559-577, doi:10.1097/00005072-198511000-00003 (1985).

2 Lacampagne, A. *et al.* Post-translational remodeling of ryanodine receptor induces calcium leak leading to Alzheimer's disease-like pathologies and cognitive deficits. *Acta Neuropathol* **134**, 749-767, doi:10.1007/s00401-017-1733-7 (2017).

3 Santulli, G. *et al.* Calcium release channel RyR2 regulates insulin release and glucose homeostasis. *J Clin Invest* **125**, 1968-1978, doi:10.1172/JCI79273 (2015).

4 Sung, H. K., Song, E., Jahng, J. W. S., Pantopoulos, K. & Sweeney, G. Iron induces insulin resistance in cardiomyocytes via regulation of oxidative stress. *Sci Rep* **9**, 4668, doi:10.1038/s41598-019-41111-6 (2019).

5 Domingo-Almenara, X. *et al.* XCMS-MRM and METLIN-MRM: a cloud library and public resource for targeted analysis of small molecules. *Nat Methods* **15**, 681-684, doi:10.1038/s41592-018-0110-3 (2018).
